# Host cells reprogram lipid droplet synthesis through YY1 to resist PRRSV infection

**DOI:** 10.1128/mbio.01549-24

**Published:** 2024-07-02

**Authors:** Zifang Zheng, Xue Ling, Yang Li, Shuang Qiao, Shuangquan Zhang, Jie Wu, Zhiqian Ma, Mingyu Li, Xuyang Guo, Zhiwei Li, Yingtong Feng, Xiao Liu, Ian G. Goodfellow, Haixue Zheng, Shuqi Xiao

**Affiliations:** 1 State Key Laboratory for Animal Disease Control and Prevention, College of Veterinary Medicine, Lanzhou University, Lanzhou Veterinary Research Institute, Chinese Academy of Agricultural Sciences, Lanzhou, Gansu, China; 2 College of Veterinary Medicine, Northwest A&F University, Yangling, Shaanxi, China; 3 Division of Virology, Department of Pathology, University of Cambridge, Cambridge, United Kingdom; University of Calgary, Calgary, Canada

**Keywords:** porcine reproductive and respiratory syndrome virus, yin yang 1, reprogram, lipid droplet, antiviral

## Abstract

Metabolism in host cells can be modulated after viral infection, favoring viral survival or clearance. Here, we report that lipid droplet (LD) synthesis in host cells can be modulated by yin yang 1 (YY1) after porcine reproductive and respiratory syndrome virus (PRRSV) infection, resulting in active antiviral activity. As a ubiquitously distributed transcription factor, there was increased expression of YY1 upon PRRSV infection both *in vitro* and *in vivo*. YY1 silencing promoted the replication of PRRSV, whereas YY1 overexpression inhibited PRRSV replication. PRRSV infection led to a marked increase in LDs, while YY1 knockout inhibited LD synthesis, and YY1 overexpression enhanced LD accumulation, indicating that YY1 reprograms PRRSV infection-induced intracellular LD synthesis. We also showed that the viral components do not colocalize with LDs during PRRSV infection, and the effect of exogenously induced LD synthesis on PRRSV replication is nearly lethal. Moreover, we demonstrated that YY1 affects the synthesis of LDs by regulating the expression of lipid metabolism genes. YY1 negatively regulates the expression of fatty acid synthase (FASN) to weaken the fatty acid synthesis pathway and positively regulates the expression of peroxisome proliferator-activated receptor gamma (PPARγ) to promote the synthesis of LDs, thus inhibiting PRRSV replication. These novel findings indicate that YY1 plays a crucial role in regulating PRRSV replication by reprogramming LD synthesis. Therefore, our study provides a novel mechanism of host resistance to PRRSV and suggests potential new antiviral strategies against PRRSV infection.

**IMPORTANCE:**

Porcine reproductive and respiratory virus (PRRSV) has caused incalculable economic damage to the global pig industry since it was first discovered in the 1980s. However, conventional vaccines do not provide satisfactory protection. It is well known that viruses are parasitic pathogens, and the completion of their replication life cycle is highly dependent on host cells. A better understanding of host resistance to PRRSV infection is essential for developing safe and effective strategies to control PRRSV. Here, we report a crucial host antiviral molecule, yin yang 1 (YY1), which is induced to be expressed upon PRRSV infection and subsequently inhibits virus replication by reprogramming lipid droplet (LD) synthesis through transcriptional regulation. Our work provides a novel antiviral mechanism against PRRSV infection and suggests that targeting YY1 could be a new strategy for controlling PRRSV.

## INTRODUCTION

Porcine reproductive and respiratory syndrome (PRRS) is a highly contagious disease caused by PRRSV infection that affects the global pig industry. It is characterized by reproductive failure of pregnant sows and respiratory diseases in pigs of all ages. Since it was first reported in the late 1980s, PRRS has resulted in considerable economic losses to the swine industry worldwide (1–3). PRRSV is an enveloped, single-positive-stranded RNA virus belonging to the order *Nidovirales* family *Arteriviridae* (4). The PRRSV genome is ~15 kb in length and contains 11 open reading frames (ORFs) encoding 8 structural proteins (Gp2a, Gp3-5, Gp5a, E, M, and N) and 16 nonstructural proteins (nsp1α, nsp1β, nsp2, nsp2TF, nsp2N, nsp3-6, nsp7α, nsp7β, and nsp8-12) (5–7). PRRSV has very restricted tropism for cells of the monocytic-macrophage lineage. Fully differentiated porcine alveolar macrophages (PAMs) are the primary host cell target for PRRSV infection (8–10). In addition, MARC-145, derived from the African green monkey kidney cell line MA-104, also supports PRRSV replication and has been widely used in studies of PRRSV *in vitro* (11–15).

Yin Yang 1 (YY1) is a ubiquitously expressed transcription factor involved in various biological and physiological processes, including DNA repair, cell proliferation, differentiation, embryonic development, and tumorigenesis (16–18). YY1 has an activation domain at the N-terminus and a repression domain at the C-terminus, which can be used as a transcriptional activator or inhibitor, depending on the context. The names “Yin” and “Yang” are concepts from ancient Chinese philosophy, reflecting the dual role of proteins in gene regulation (19, 20). Mice lacking YY1 exhibit lethality during embryo implantation, highlighting its important role in development (21). It has been reported that YY1 is associated with virus production (22, 23). YY1 binds to DNA sequences in the human immunodeficiency virus type 1 (HIV-1) long terminal repeat (LTR), recruiting histone deacetylase 1 and 2 (HDAC1/2) to promote the deacetylation of histones near the HIV-1 LTR, resulting in the prolonged latency of HIV-1 (24, 25). Interestingly, Wang et al. reported that YY1 can act as an effective transactivator of the human T lymphotropic virus type 1 (HTLV-1) LTR and that it binds to viral RNA instead of through classical DNA–transcription factor interactions, which is in sharp contrast to its ability to inhibit other retroviral genomes (26). YY1 has been reported to be highly expressed in many cancer tissues, promoting tumor cell proliferation and angiogenesis in a p53-dependent or p53-independent manner (27–30). Metabolic reprogramming is a characteristic of tumor cells. Recent studies have shown that YY1 is also involved in glycometabolism reprogramming, lipid metabolism, and bile acid metabolism in hepatocellular carcinoma (HCC) cells and is strongly related to the development of liver diseases (31–33). We found that there was increased expression of YY1 upon PRRSV infection. However, whether YY1 regulates PRRSV replication remains unknown.

Lipid droplets (LDs) are dynamic organelles, and cells store fat as LDs (34, 35). Lipid esters are deposited intracellularly, are surrounded by a monolayer of phospholipids, and are separated from the hydrophilic cytoplasmic environment by a layer of structural proteins (36, 37). Lipid metabolism reprogramming occurs in various tumor cells and is a crucial factor driving cancer progression (38–41). Due to the lack of independent metabolic capacity, viruses must rely on host cells to complete their life cycle. Recent studies have shown that LDs play an important role in the infection process of various viruses. In particular, *Flaviviridae* viruses can consume LDs during infection to manipulate lipid metabolism in host cells to obtain the energy needed for self-replication. They also use LDs as viral replication and assembly platforms to promote their proliferation. For example, dengue virus (DENV) infection results in the release of free fatty acids (FFAs) from LDs through an autophagy-dependent process, leading to an increase in cellular β-oxidation, which generates ATP for virus replication (42). Hepatitis C virus (HCV) assembly and production rely on LDs. HCV infection induces the expression of sterol regulatory element-binding protein (SREBP), which is the major transcription factor for lipogenic genes, leading to increased LD formation to facilitate viral assembly (43). Studies have shown that FFAs are also required for PRRSV replication (44, 45). Here, we report that YY1 is highly expressed in lung tissue and PAMs after PRRSV infection and significantly inhibits PRRSV replication both *in vivo* and *in vitro*. YY1 reprograms intracellular LD synthesis by regulating the expression of fatty acid synthase (FASN) and peroxisome proliferator-activated receptor gamma (PPARγ), thereby inhibiting PRRSV replication.

Our study reveals a novel mechanism by which host cells actively resist PRRSV infection and may be a promising therapeutic target for treating PRRS.

## RESULTS

### YY1 inhibits PRRSV replication *in vitro*

**Fig 1:**
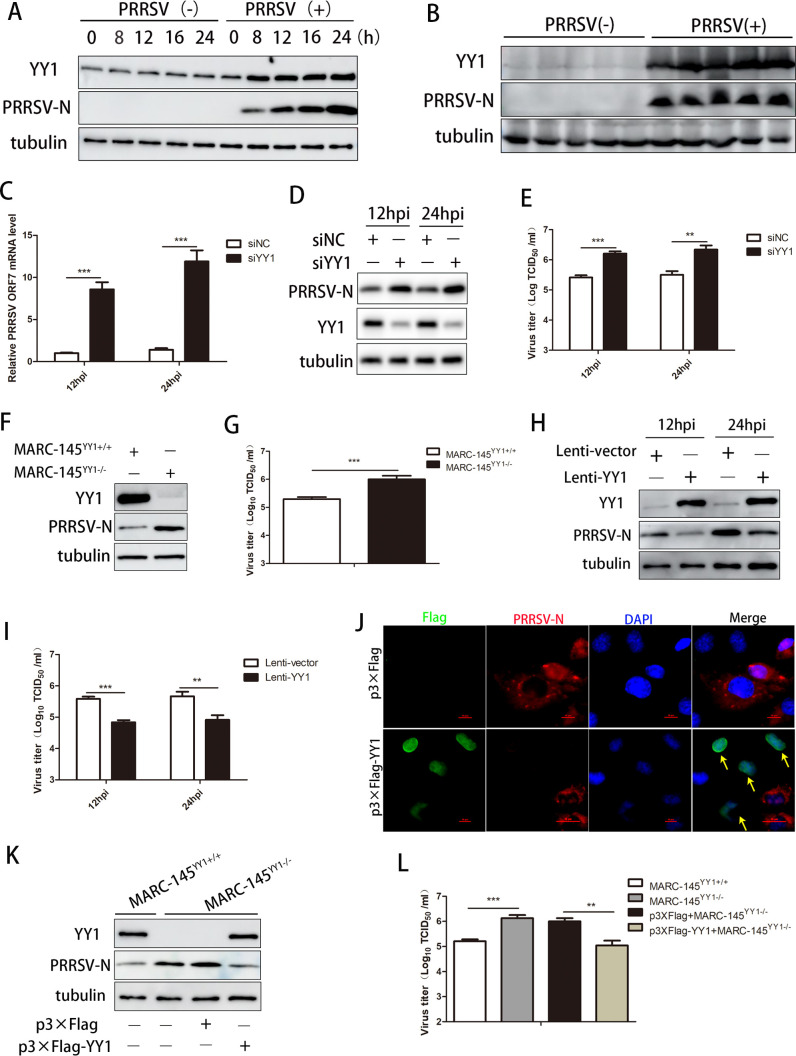
YY1 inhibits PRRSV replication *in vitro*. (**A**) PAMs were infected or not infected with PRRSV (MOI = 1) for the indicated periods, and the cells were harvested to detect the expression of YY1 by Western blotting. Tubulin served as an internal control, and the PRRSV N protein was used as an infection indicator. (**B**) The lung tissues of five piglets infected with PRRSV and five piglets not infected with PRRSV were collected and crushed, and the expression of YY1 was detected by Western blotting. Tubulin served as an internal control, and the PRRSV N protein was used as an infection indicator. (**C–E**) PAMs were transfected with si-NC or si-YY1 at a concentration of 50 nM for 24 h, followed by infection with PRRSV (MOI = 1) for the indicated periods. Cells and supernatants were harvested to determine (**C**) PRRSV ORF7 mRNA expression, (**D**) YY1 and PRRSV N protein expression, tubulin served as an internal control, and (**E**) supernatant virus titer. (**F and G**) YY1^+/+^ and YY1^−/−^ MARC-145 cells were infected with PRRSV (MOI = 1) for 36 h, the cells and supernatants were harvested to determine (**F**) YY1 and PRRSV N protein expression, tubulin served as an internal control, (**G**) supernatant virus titer. (**H and I**) PAMs were infected with recombinant lentivirus expressing YY1 or control lentivirus, followed by infection with PRRSV (MOI = 1) for the indicated periods. Cells and supernatants were harvested to determine (**H**) YY1 and PRRSV N protein expression, tubulin served as an internal control, and (**I**) the supernatant virus titer. (**J**) MARC-145 cells were transfected with p3×Flag (vector) or p3×Flag-YY1 for 24 h, followed by infection with PRRSV (MOI = 1) for 36 h. The cells were harvested to determine the location of the PRRSV-N protein and YY1 by immunofluorescence analysis. (**K and L**) YY1^−/−^ MARC-145 cells were transfected with p3×Flag (vector) or p3×Flag-YY1 for 24 h, followed by infection with PRRSV (MOI = 1) for 36 h. The cells and supernatants were harvested to determine (**K**) YY1 and PRRSV N protein expression, and tubulin served as an internal control, and (**L**) supernatant virus titer. *P* values were calculated using Student’s *t*-test. **P < 0.01; ***P < 0.001.

To investigate the role of YY1 during PRRSV infection, PAMs, the primary host cell target for PRRSV infection, were infected with PRRSV GD-HD at different times to detect the expression of YY1. As shown in Fig. 1A, the PRRSV nucleocapsid (N) protein was used as an infection indicator, and the protein level of YY1 increased with PRRSV infection *in vitro*. We further collected the lungs of five piglets infected with PRRSV and five piglets not infected with PRRSV and detected high expression of the YY1 protein in PRRSV-infected piglets (Fig. 1B). To assess the impact of ablating YY1 expression on viral expression, we transfected specific siRNAs into PAMs and found that knockdown of YY1 significantly increased PRRSV ORF7 mRNA levels at 12 and 24 h following viral infection (Fig. 1C). Moreover, the PRRSV N protein level and virus titer in the YY1 knockdown group were greater than those in the NC group (Fig. 1D and E). Subsequently, sgRNA was specifically designed to target the YY1 gene in MARC-145 cells (Fig. S1A), and we successfully constructed YY1 knockout cell lines (Fig. S1B and C). The experimental results showed that the PRRSV N protein level and progeny virus titer were significantly increased with YY1 knockout (Fig. 1F and G). The fluorescence microscopy results also showed that the PRRSV-mCherry strain could replicate better in YY1 knockout cells (Fig. S1D).

To confirm the negative role of YY1 in PRRSV replication, PAMs were infected with recombinant lentivirus expressing YY1 or control lentivirus and then infected with PRRSV. As expected, the overexpression of YY1 significantly inhibited viral replication, which was reflected in the expression level of the PRRSV N protein and the viral titer (Fig. 1H and I). Similarly, overexpression of YY1 in MARC-145 cells using eukaryotic plasmids also inhibited PRRSV replication (Fig. S1E and F). We also confirmed the antiviral effect of YY1 by immunofluorescence using confocal microscopy, and the location of the PRRSV-N protein was not detected in YY1-overexpressing positive cells (Fig. 1J). Finally, we transfected a YY1 eukaryotic expression plasmid into knockout cell lines and found that the overexpression of YY1 reversed the ability of YY1 knockout to promote PRRSV replication (Fig. 1K and L). Here, we demonstrated that YY1 is a host restriction factor in PRRSV replication.

### YY1 inhibits PRRSV replication in piglets

**Fig 2:**
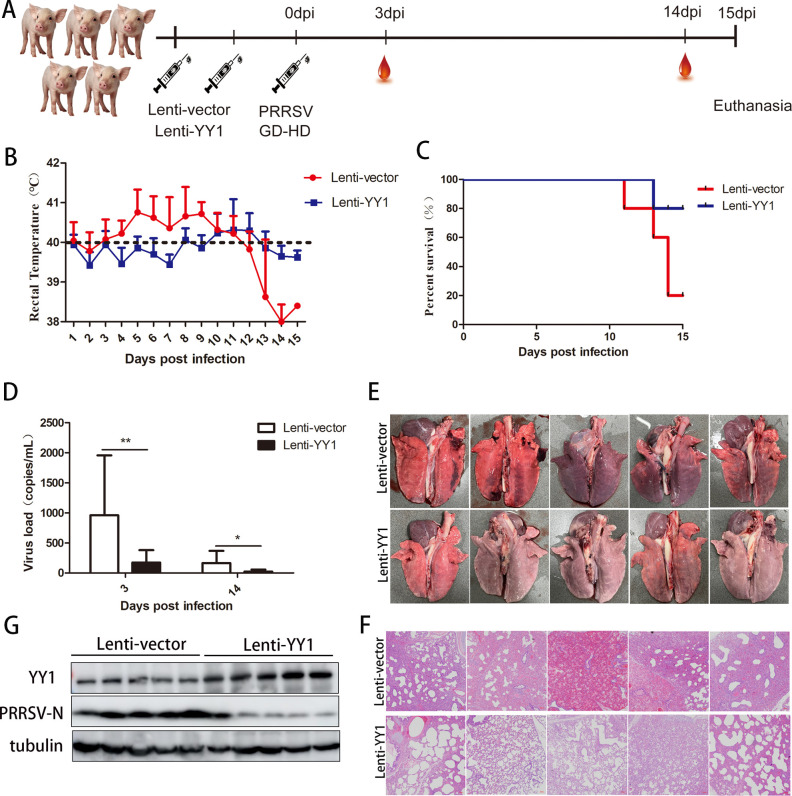
YY1 inhibits PRRSV replication *in vivo*. (**A**) Flow chart of the animal experiments. Ten piglets were randomly divided into two groups, with five piglets in each group. The piglets were infected with recombinant lentivirus expressing YY1 (experimental group) or control lentivirus (NC group) and reinfected 2 days later at the same dose. Two days after lentivirus reinfection, each piglet was infected with PRRSV GD-HD. Serum samples were collected from each group on 3 and 14 days after PRRSV infection, and the remaining piglets were euthanized on 15 days. (**B**) The rectal temperature of the piglets was recorded daily. (**C**) The survival rate of the piglets in each group. (**D**) The viral load in the serum was detected by real-time qPCR. A recombinant plasmid containing the PRRSV ORF7 gene was used to construct a standard curve, and the viral load in each serum sample was calculated according to the standard curve. (**E**) Pathological changes in the lung surface were observed. The extracted piglet lungs were washed with PBS and placed on clean ground for filming. (**F**) Lung tissue sections from piglets in the two groups were stained with hematoxylin and eosin (HE). (**G**) The lungs of five piglets in the experimental group and five piglets in the control group were collected and crushed, and the protein expression of YY1 and PRRSV N was detected by Western blotting. Tubulin served as an internal control. *P* values were calculated using student’s *t*-test. *P < 0.05; **P < 0.01.

To further validate the antiviral function of YY1 *in vivo*, 10 piglets were randomly divided into two groups, with 5 piglets in each group. The piglets were infected with recombinant lentivirus expressing YY1 (experimental group) or control lentivirus (NC group) and reinfected 2 days later at the same dose. Two days after the completion of lentivirus reinfection, each piglet was infected with PRRSV GD-HD, and serum samples from the piglets in each group were collected on 3 and 14 days after PRRSV infection (Fig. 2A). All the piglets were carefully monitored until the end of the experiment to observe the clinical signs of PRRS. The NC group showed obvious clinical signs, such as inappetence, diarrhea, and purple surfaces on the ears, limbs, and abdomen after being challenged with PRRSV. As shown in Fig. 2B, the NC group exhibited fever beginning at 3 days postinfection (dpi) that lasted after 9 days, with the highest average temperature reaching 40.76°C at 5 dpi, and the temperature of one piglet even reached 41.5°C. In the experimental group, the fever started at 10 dpi and lasted for 3 days, with the highest average temperature reaching 40.32°C at 11 dpi. During the experiment, four piglets died in the NC group at 11, 13, and 14 dpi, while only one piglet died in the experimental group at 13 dpi (Fig. 2C). The serum viral load of the NC group was also significantly greater than that of the experimental group (Fig. 2D). At 15 dpi, the remaining piglets were euthanized, and lung tissues were collected. More severe interstitial pneumonia and pulmonary hemorrhage occurred in the lungs of piglets in the NC group (Fig. 2E and F). Finally, we detected PRRSV replication in the lungs and found that YY1 was overexpressed and significantly inhibited the expression of the PRRSV N protein (Fig. 2G). These results indicate that YY1 inhibits PRRSV replication *in vivo*.

### IFN-β regulated by YY1 is not the main mechanism to inhibit PRRSV replication

**Fig 3:**
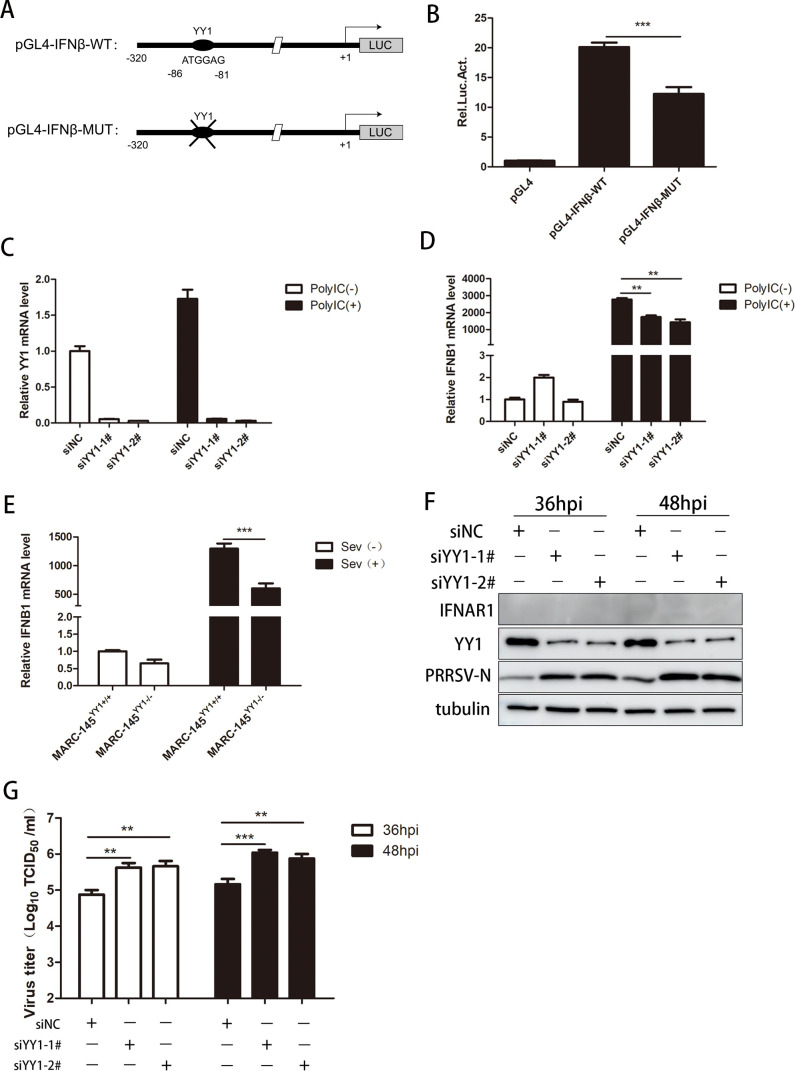
The regulation of IFN-β by YY1 is not the main mechanism by which it inhibits PRRSV replication. (**A**) Schematic representation of the IFN-β promoter. The binding site of YY1 was annotated and inserted into the dual-luciferase reporter plasmid pGL4 with the YY1 consensus site (pGL4-IFNβ-WT) or without the YY1 consensus site (pGL4-IFNβ-MUT). (**B**) MARC-145 cells were transfected with the indicated plasmids and phRL-TK. The cells were collected to detect the promoter activity of IFNβ-WT and IFNβ-MUT. (**C and D**) MARC-145 cells were transfected with si-NC or si-YY1 at a concentration of 50 nM for 24 h, followed by transfection with poly(I:C) for 12 h, and the cells were harvested to detect (**C**) YY1 mRNA expression and (**D**) IFN-β mRNA expression. (**E**) YY1^+/+^ and YY1^−/−^ MARC-145 cells were infected with Sev for 12 h, and the cells were harvested to detect IFN-β mRNA expression. (**F and G**) IFNAR1^−/−^ MARC-145 cells were transfected with si-NC or si-YY1 at a concentration of 50 nM for 24 h, followed by infection with PRRSV (MOI = 1) for 36 and 48 h. The cells and supernatants were harvested to determine (**F**) IFNAR1, YY1, and PRRSV N protein expression, and tubulin served as an internal control, and (**G**) supernatant virus titer. *P* values were calculated using Student’s *t*-test. **P < 0.01; ***P < 0.001.

Previous studies have shown that YY1 binds to the promoter region of IFN-β for transcriptional regulation (46), and there is no doubt that IFN-β is lethal to PRRSV proliferation. Exogenous addition of IFN-β significantly decreased the PRRSV N protein and progeny virus titers (Fig. S2A and B). To explore the molecular mechanism by which YY1 inhibits PRRSV replication, we first speculated whether YY1 deficiency affects IFN-β in host cells, thus creating favorable conditions for PRRSV replication. First, the IFN-β promoter plasmid pGL4-IFNβ-WT was constructed, the binding site of YY1 was predicted in this region, and the site-deletion mutant plasmid pGL4-IFNβ-MUT was constructed according to this site (Fig. 3A). As shown in Fig. 3B, the promoter activity of IFN-β was significantly downregulated in the absence of the YY1 binding site, suggesting a positive regulatory effect of YY1 on IFN-β. Then, specific siRNAs were used to reduce the expression of YY1 (Fig. 3C), followed by incubation with poly(I:C), a double-stranded RNA simulant that is the classic type I IFN inducer. YY1 knockdown inhibited the expression of IFN-β induced by poly(I:C) (Fig. 3D). Consistently, the expression levels of IFN-β induced by Sendai virus (SeV), another effective inducer of type I IFN, were significantly lower in YY1 knockout cells than in control cells (Fig. 3E). Given that YY1 deficiency results in decreased expression of IFN-β after poly(I:C) treatment or infection with SeV, we hypothesized that this effect would also occur during PRRSV infection and, thus, facilitate viral replication. To further test this hypothesis, sgRNA was specifically designed to target the IFNAR1 gene (Fig. S2C), which is the receptor for IFN-β-mediated activation of the innate immune pathway. We generated IFNAR1 knockout MARC-145 cell lines (Fig. S2D and E) and then transfected them with specific siRNAs targeting YY1. Surprisingly, we found that YY1 knockdown mediated an increase in PRRSV replication even under IFNAR1 knockout conditions (Fig. 3F and G). These data indicate that YY1-regulated IFN-β is not the main mechanism inhibiting PRRSV replication.

### YY1 reprograms the synthesis of intracellular lipid droplets to perform antiviral functions

**Fig 4:**
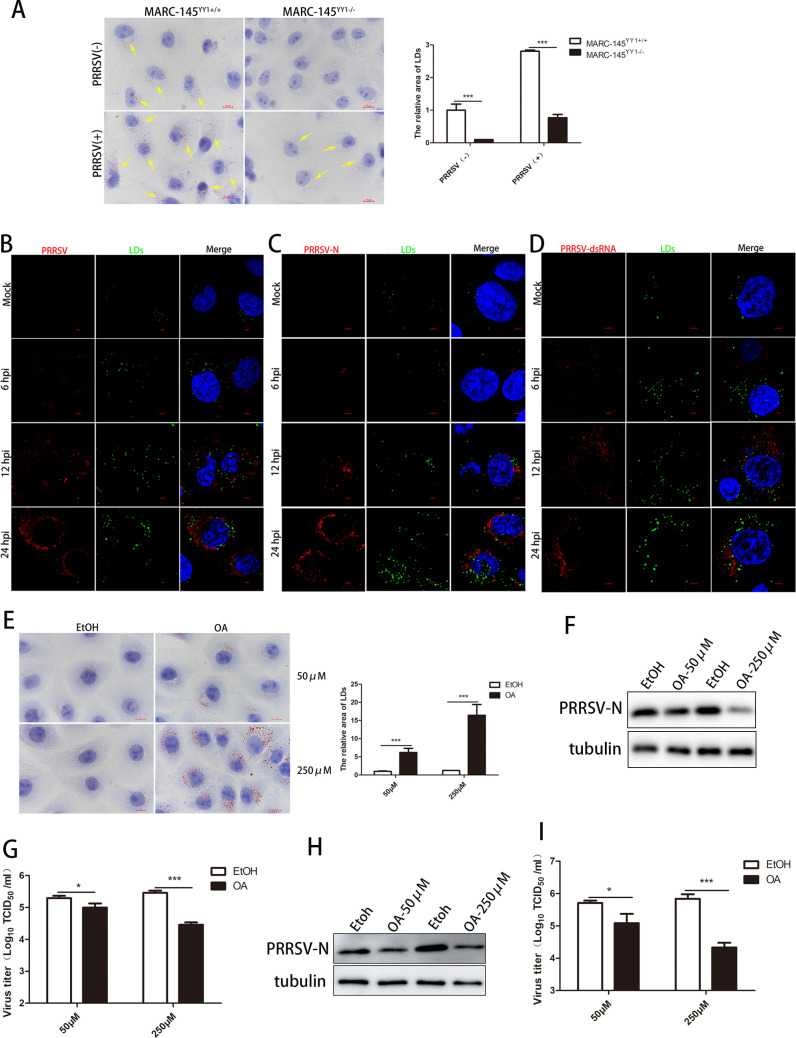
YY1 reprograms the synthesis of intracellular lipid droplets to exert antiviral effects. (**A**) YY1^+/+^ and YY1^−/−^ MARC-145 cells were fixed, the LDs were stained with Oil Red O, and the nuclei were counterstained with hematoxylin. (**B–D**) MARC-145 cells were infected with PRRSV (MOI = 0.1) for 0, 6, 12, and 24 h, and the LDs were stained with a BODIPY probe (green). The viruses were labeled with PRRSV-positive serum (red), PRRSV nucleocapsid protein-specific antibody (red), and dsRNA antibody (red). Confocal microscopy was used to photograph and analyze (**B**) the colocalization between virus particles and LDs, (**C**) the colocalization between PRRSV nucleocapsid protein and LDs, and (**D**) the colocalization between PRRSV dsRNA and LDs. (**E**) MARC-145 cells were incubated with 50 or 250 µM oleic acid (dissolved in absolute ethanol) for 24 h, and the cells were fixed and stained with Oil Red O. The nuclei were counterstained with hematoxylin, and the number of lipid droplets in the cells was analyzed using a 100× oil lens. (**F and G**) MARC-145 cells were infected with PRRSV (MOI = 1) for 1 h, and the virus was removed and incubated with 50 or 250 µM oleic acid for 24 h. The cells and supernatants were harvested to determine (**F**) PRRSV N protein expression, tubulin served as an internal control, and (**G**) the supernatant virus titer. (**H and I**) PAMs were infected with PRRSV (MOI = 1) for 1 h, and the virus was removed and incubated with 50 or 250 µM oleic acid for 24 h. Cells and supernatants were harvested to determine (**H**) PRRSV N protein expression, tubulin served as an internal control, and (**I**) supernatant virus titer. *P* values were calculated using Student’s *t*-test. *P < 0.05; ***P < 0.001.

As the energy storage center in the cell, LDs play an important role in energy competition between viruses and hosts after virus infection. Recent studies have shown that FFAs, the products of lipid metabolism, play a vital role in PRRSV replication (44, 45). YY1 reportedly facilitates hepatocellular carcinoma cell (HCC) lipid metabolism and tumor progression by inhibiting PGC-1β-induced fatty acid oxidation (32). Therefore, we hypothesized that YY1 affects PRRSV replication by changing the intracellular lipid metabolism process. First, Oil Red O and BODIPY staining were used to detect changes in intracellular LDs. We found that PRRSV infection increased the number of LDs in cells, and YY1 knockout significantly inhibited intracellular LD synthesis regardless of the presence or absence of PRRSV infection (Fig. 4A; Fig. S3A). Then, recombinant cell lines stably expressing YY1 were established (Fig. S3B). In contrast, the overexpression of YY1 enhanced LD accumulation (Fig. S3C and D). We further transfected a YY1 eukaryotic expression plasmid into knockout cell lines and found that YY1 overexpression restored intracellular lipid droplet synthesis (Fig. S3E).

Viral infections (such as those caused by DENV and PRRSV) activate the autophagy pathway and promote the decomposition of LDs to free fatty acids that provide energy for viral replication (42, 44). However, DENV relies on capsid proteins to attach to lipid droplet surfaces to assemble progeny virions (47). To determine whether LDs are required for PRRSV assembly, we labeled the virus with PRRSV-positive serum and a PRRSV nucleocapsid protein-specific antibody, stained the LDs with a BODIPY probe, and analyzed the colocalization of the viral components and LDs by confocal microscopy. Time-course studies revealed that the virus particles and nucleocapsid proteins did not colocalize with LDs during PRRSV infection (Fig. 4B and C), suggesting that the assembly process of PRRSV does not proceed through LDs. The replication process of the PRRSV genome begins with a replication and transcription complex (RTC) composed of nonstructural proteins. We found that nsp2, nsp9, and nsp10, which are important components of the PRRSV RTC, do not colocalize with LDs (Fig. S4A). Moreover, the double-stranded RNA produced during PRRSV replication did not colocalize with intracellular LDs (Fig. 4D), suggesting that the replication process of the PRRSV genome does not occur on the surface of LDs. Next, exogenous oleic acid (OA) was added to the culture medium for lipid supplementation to explore the effect of LDs on PRRSV replication. As expected, the two concentrations of OA promoted the synthesis of intracellular LDs to varying degrees and did not affect cell activity (Fig. 4E; Fig. S4B). The experimental results showed that the PRRSV N protein level and progeny virus titer were significantly decreased with OA treatment in MARC-145 cells (Fig. 4F and G). Similarly, exogenously added OA inhibited PRRSV replication in PAMs (Fig. 4H and I). These results indicate that increased intracellular LD is likely a strategy for the host to resist viral infection and that YY1 plays an important role in this process.

### YY1 reprograms the synthesis of LDs by regulating lipid metabolism genes

**Fig 5:**
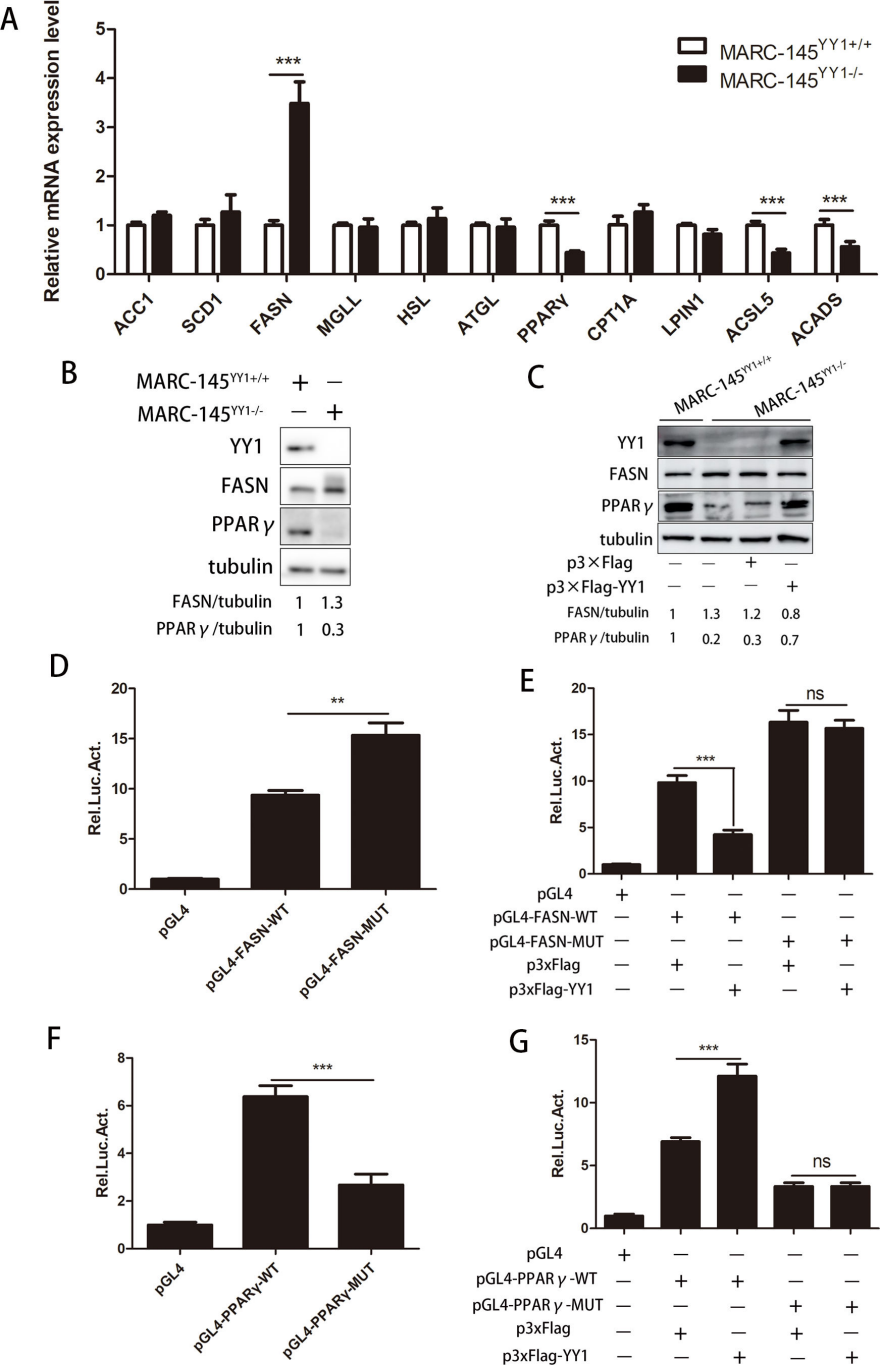
YY1 regulates the expression of the lipid metabolism genes FASN and PPARγ. (**A**) mRNA expression levels of various lipid metabolism-associated factors in YY1^+/+^ and YY1^−/−^ MARC-145 cells, as determined using reverse transcriptase PCR (RT-qPCR). (**B**) Western blot analysis of FASN and PPARγ protein expression in YY1^+/+^ and YY1^−/−^ MARC-145 cells, tubulin served as an internal control. (**C**) YY1^−/−^ MARC-145 cells were transfected with p3×Flag (vector) or p3×Flag-YY1 for 24 h, and the protein expression levels of YY1, FASN, and PPARγ in YY1^+/+^ and YY1^−/−^ MARC-145 cells were measured, tubulin served as an internal control. (**D and E**) MARC-145 cells were transfected with the indicated plasmids and phRL-TK. The cells were collected to detect the promoter activity of FASN-WT and FSAN-MUT. (**F and G**) MARC-145 cells were transfected with the indicated plasmids and phRL-TK. The cells were collected to detect the promoter activity of PPARγ-WT and PPARγ-MUT. *P* values were calculated using Student’s *t*-test. ns, not significant; **P < 0.01; ***P < 0.001.

As a transcription factor, YY1 likely directly regulates the expression of lipid metabolism genes, thus affecting the synthesis of LDs (32, 48, 49). To verify this, we detected the expression of several important lipid metabolism genes in YY1-knockout and YY1-overexpressing cells (32). As shown in Fig. 5A, among the genes whose mRNA expression levels were affected by YY1 knockout, FASN was significantly increased, while PPARγ, ACSL5, and ACADS were decreased. Consistent with the results of YY1 knockout, YY1 overexpression significantly suppressed FASN expression and promoted PPARγ expression but did not affect ACSL5 or ACADS expression (Fig. S5A). The changes in the protein expression levels of FASN and PPARγ were consistent with the changes in the mRNA levels (Fig. 5B; Fig. S5B). Moreover, the regulatory effect of YY1 on FASN and PPARγ expression was also established in PAMs (Fig. S5C and D). Finally, we transfected a YY1 eukaryotic expression plasmid into knockout cell lines and found that the overexpression of YY1 could reverse the regulatory effect of YY1 knockout on FASN and PPARγ protein expression (Fig. 5C).

FASN is an important enzyme in fatty acid synthesis (50), while PPARγ promotes fatty acid uptake, triglyceride formation, and lipid storage (51). YY1 inhibited FASN expression but promoted PPARγ expression, suggesting that YY1 affects the synthesis of intracellular LDs by regulating lipid metabolism genes. To further verify whether YY1 directly regulates FASN and PPARγ expression, we analyzed the promoter sequences of FASN and PPARγ and found YY1-binding sites in their promoter regions. Next, we constructed FASN and PPARγ promoter reporter plasmids containing the YY1-binding site and FASN and PPARγ promoter reporter plasmids lacking the YY1-binding site (Fig. S5E and F). The luciferase reporter assay showed that the wild-type FASN and PPARγ promoter plasmids had stronger activity (Fig. 5D and F). However, after the YY1-binding site was deleted, the mutant promoter activity of FASN further increased, while the mutant promoter activity of PPARγ significantly decreased (Fig. 5D and F). In addition, cotransfection with the eukaryotic YY1 expression plasmid significantly reduced FASN-WT promoter activity and increased PPARγ-WT promoter activity (Fig. 5E and G). After the binding site of YY1 was deleted, the regulatory effect of YY1 on FASN-MUT and PPARγ-MUT promoter activity decreased (Fig. 5E and G). These results indicate that YY1 directly regulates the promoter activity of FASN and PPARγ, thus manipulating FASN and PPARγ expression.

### YY1 inhibits PRRSV replication by regulating FASN-mediated fatty acid synthesis

**Fig 6:**
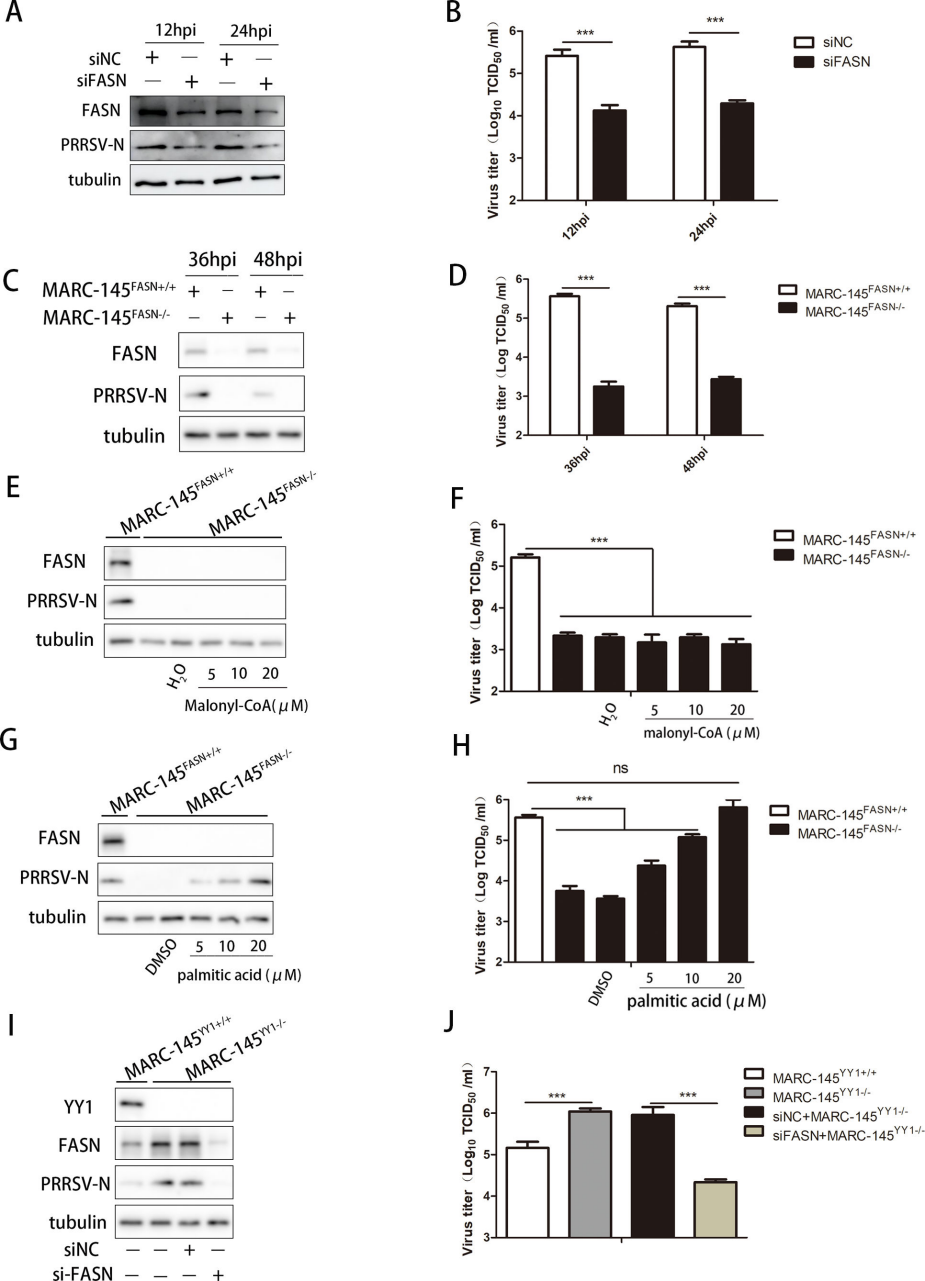
YY1 affects PRRSV replication by regulating FASN-mediated fatty acid synthesis. (**A and B**) PAMs were transfected with si-NC or si-FASN at a concentration of 50 nM for 24 h, followed by infection with PRRSV (MOI = 1) for the indicated periods. Cells and supernatants were harvested to determine (**A**) FASN and PRRSV N protein expression, tubulin served as an internal control, and (**B**) supernatant virus titer. (**C and D**) FASN^+/+^ and FASN^−/−^ MARC-145 cells were infected with PRRSV (MOI = 1) for the indicated periods, and the cells and supernatants were harvested to determine (**C**) FASN and PRRSV N protein expression, tubulin served as an internal control, and (**D**) supernatant virus titers. (**E and F**) FASN^+/+^ and FASN^−/−^ MARC-145 cells were infected with PRRSV (MOI = 1) for 1 h, the virus was removed and incubated with different concentrations of malonyl-CoA (dissolved in distilled water) for 36 h. Cells and supernatants were harvested to determine (**E**) FASN and PRRSV N protein expression, tubulin served as an internal control, and (**F**) supernatant virus titer. (**G and H**) FASN^+/+^ and FASN^−/−^ MARC-145 cells were infected with PRRSV (MOI = 1) for 1 h, the virus was removed and incubated with different concentrations of palmitic acid (dissolved in DMSO) for 36 h. Cells and supernatants were harvested to determine (**G**) FASN and PRRSV N protein expression, and tubulin served as an internal control, and (**H**) supernatant virus titers. (**I and J**) YY1^+/+^ and YY1^−/−^ MARC-145 cells were transfected with si-NC or si-FASN at a concentration of 50 nM for 24 h, followed by infection with PRRSV (MOI = 1) for 36 h. The cells and supernatants were harvested to determine (**I**) YY1, FASN, and PRRSV N protein expression, tubulin serving as an internal control, and (**J**) supernatant virus titers. *P* values were calculated using Student’s *t*-test. ns, not significant; ***P < 0.001.

FASN is a critical enzyme in the fatty acid synthesis process, and its activity is essential for synthesizing the cell membrane and different lipid substrates (50). Based on the regulatory effect of YY1 on FASN, we speculate that FASN also affects the replication process of PRRSV. To verify this, we transfected specific siRNAs into PAMs. We found that FASN knockdown significantly reduced the expression level of the PRRSV N protein (Fig. 6A) and decreased the viral titer in the supernatant (Fig. 6B). Subsequently, sgRNA was specifically designed to target the FASN gene in MARC-145 cells (Fig. S6A). We successfully constructed FASN knockout cell lines (Fig. S6B and C). Similarly, knocking out FASN in MARC-145 cells significantly inhibited PRRSV replication (Fig. 6C and D). In the process of fatty acid synthesis (45), acetyl-CoA is metabolized to malonyl-CoA via acetyl-CoA carboxylase 1 (ACC1), which is then transformed into palmitic acid by FASN. Palmitic acid is prolonged and desaturated to form long-chain unsaturated fatty acids (Fig. S6D).

Furthermore, FASN knockout inhibited PRRSV replication via the FASN-mediated fatty acid synthesis pathway. FASN knockout cells were infected with PRRSV and then incubated with exogenous malonyl-CoA and palmitic acid, and the corresponding concentrations of malonyl-CoA and palmitic acid did not affect cell viability (Fig. S6E and F). Figure 6E and F show that exogenous malonyl-CoA did not promote PRRSV replication in FASN knockout cells. However, palmitic acid reversed FASN-mediated inhibition of PRRSV replication in a dose-dependent manner (Fig. 6G and H), suggesting that FASN-mediated fatty acid synthesis is essential for PRRSV replication. Finally, we examined whether YY1 regulates FASN-mediated fatty acid synthesis, which inhibits PRRSV replication. siRNAs specific for FASN were transfected into YY1 knockout cells, which were subsequently infected with PRRSV. According to our results, the knockout of YY1 increased FASN protein expression and promoted PRRSV replication, and the silencing of FASN reversed the YY1 knockout-mediated promotion of PRRSV replication (Fig. 6I and J). These data show that YY1 affects PRRSV replication by regulating FASN-mediated fatty acid synthesis.

### YY1 inhibits PRRSV replication by regulating the PPARγ-mediated LD synthesis pathway

**Fig 7:**
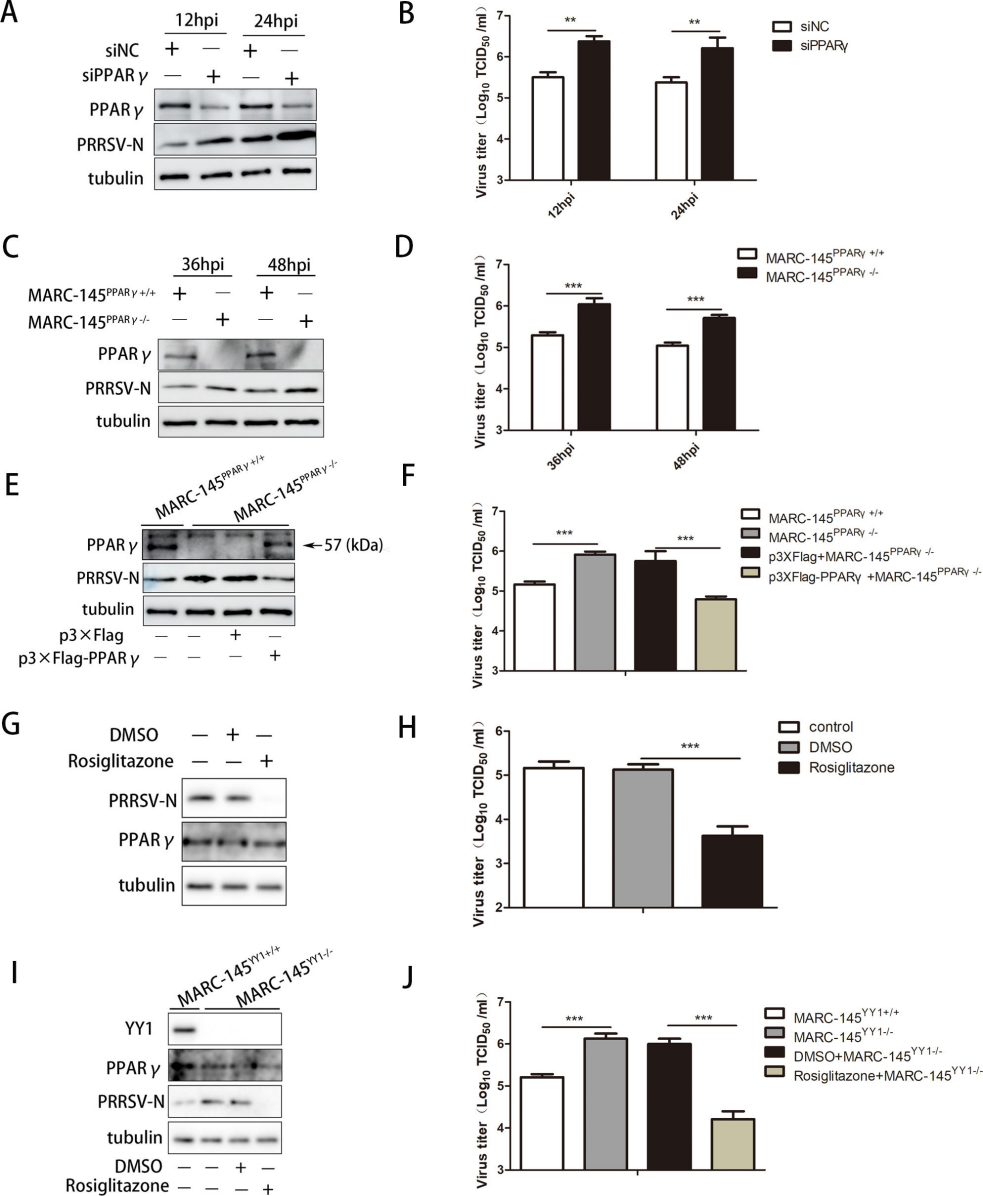
YY1 affects PRRSV replication by regulating PPARγ-mediated LD synthesis. (**A and B**) PAMs were transfected with si-NC or si-PPARγ at a concentration of 50 nM for 24 h, followed by infection with PRRSV (MOI = 1) for the indicated periods. Cells and supernatants were harvested to determine (**A**) PPARγ and PRRSV N protein expression, tubulin served as an internal control, and (**B**) supernatant virus titer. (**C and D**) PPARγ^+/+^ and PPARγ^−/−^ MARC-145 cells were infected with PRRSV (MOI = 1) for the indicated periods, and the cells and supernatants were harvested to determine (**C**) PPARγ and PRRSV N protein expression, tubulin served as an internal control, and (**D**) the supernatant virus titer. (**E and F**) PPARγ^−/−^ MARC-145 cells were transfected with p3×Flag (vector) or p3×Flag-PPARγ for 24 h, followed by infection with PRRSV (MOI = 1) for 36 h. The cells and supernatants were harvested to determine (**E**) PPARγ and PRRSV N protein expression, tubulin served as an internal control, and (**F**) the supernatant virus titer. (**G and H**) MARC-145 cells were infected with PRRSV (MOI = 1) for 1 h, and the virus was removed and incubated with 10 µM rosiglitazone for 36 h. The cells and supernatants were harvested to determine (**G**) PRRSV N protein expression, tubulin served as an internal control, and (**H**) supernatant virus titers. (**I and J**) YY1^+/+^ and YY1^−/−^ MARC-145 cells were infected with PRRSV (MOI = 1) for 1 h, the virus was removed, and the cells were incubated with 10 µM rosiglitazone for 36 h. The cells and supernatants were harvested to determine (**I**) YY1 and PRRSV N protein expression, tubulin served as an internal control, and (**J**) supernatant virus titers. *P* values were calculated using Student’s *t*-test. **P < 0.01; ***P < 0.001.

PPARγ is most highly expressed in white adipose tissue, where it is a master regulator of adipogenesis (52). Based on the regulatory effect of YY1 on PPARγ, we next explored the effect of PPARγ on PRRSV replication. First, specific PPARγ siRNAs were transfected into PAMs, and we found that PPARγ knockdown significantly increased the protein expression level of PRRSV N (Fig. 7A) and increased the viral titer in the supernatant (Fig. 7B). Subsequently, sgRNA was specifically designed to target the PPARγ gene in MARC-145 cells (Fig. S7A). We successfully constructed PPARγ knockout cell lines (Fig. S7B and C), and PPRAγ knockout inhibited the synthesis of intracellular LDs (Fig. S7D). Similarly, knocking out PPARγ in MARC-145 cells significantly promoted PRRSV replication (Fig. 7C and D). To confirm the negative role of PPARγ in PRRSV replication, we transfected a PPARγ eukaryotic expression plasmid into knockout cell lines and found that PPARγ overexpression reversed the ability of PPARγ knockout to promote PRRSV replication (Fig. 7E and F). Rosiglitazone, a potent and selective activator of PPARγ, was used to determine the negative regulatory effect of PPARγ on PRRSV replication. As expected, it promoted the synthesis of intracellular LDs (Fig. S7E) and significantly inhibited PRRSV replication (Fig. 7G and H). Finally, we examined whether YY1 regulates PPARγ-mediated LD synthesis, which inhibits PRRSV replication. YY1 knockout cells were infected with PRRSV, followed by incubation with rosiglitazone. As shown in Fig. 7I and J, knockout of YY1 reduced the protein expression level of PPARγ and promoted PRRSV replication, and activation of PPARγ reversed the YY1 knockout-mediated promotion of PRRSV replication. These data show that YY1 affects PRRSV replication by regulating PPARγ-mediated LD synthesis.

## DISCUSSION

Cellular metabolism involves various biological processes, including interactions between viruses and hosts. Viral infection induces metabolic dysregulation in host cells, creating favorable conditions for self-replication. Conversely, host cells are also actively reprogrammed metabolically, inhibiting viral replication for virus clearance (53–56). Multiple regulators participate in these changes during virus-host interactions. Understanding these dynamic processes within host cells is crucial for identifying potential antiviral therapy targets. In this study, we report the function of the host molecule YY1 in PRRSV infection, and compared with the positive regulation of IFN-β, YY1 exerts antiviral effects mainly through the reprogramming of intracellular LD synthesis.

YY1 has been reported to bind to the promoter region of IFN-β in mice and has dual activation and inhibitory effects on IFN-β promoter activity depending on its binding site and time after viral infection (46). Here, we found that the promoter activity of IFN-β was significantly downregulated after the deletion of the YY1-binding site, and YY1 deficiency suppressed the expression of IFN-β induced by poly(I:C) and Sev, which may explain the mechanism by which YY1 regulates PRRSV replication. Surprisingly, the increase in PRRSV replication mediated by YY1 knockdown still occurred in the presence of IFNAR1 knockout (Fig. 3). Thus, the attenuated IFN-β response induced by YY1 knockout is not the main cause of the YY1-mediated suppression of PRRSV replication, which is the first unexpected finding of the study.

It has been reported that viral infections reprogram the lipid metabolism of host cells to create favorable conditions for the generation of progeny viruses (57, 58). The HCV core protein forms the viral capsid and targets LDs through its D2 domain. The binding strength is critical for determining viral assembly efficiency (59). SARS-CoV-2 infection also induces LD synthesis, with both virus particles and dsRNA localized on the LD surface and using LDs as a platform for viral replication (60). PRRSV activates the autophagy pathway by downregulating the expression of N-myc downstream-regulated gene 1 (NDRG1), inducing the degradation of lipid droplets into FFAs to obtain the energy required for viral self-replication (44). LDs play multiple and integral roles in the life cycle of viruses. However, LDs do not simply serve as a convenient site for virus replication. Although HCV can assemble progeny viruses on the surface of LDs, some host proteins localized on the surface of LDs have been reported to have anti-HCV activity (61). By continuing to explore the antiviral mechanism of YY1, we found that YY1 promoted intracellular LD synthesis and that the number of intracellular LDs also increased significantly after PRRSV infection (Fig. 4A; Fig. S3), this finding is another interesting aspect of this study. Suppose the increased LD number is the result of PRRSV induction. In that case, it can directly hijack the raw material FFAs synthesized by LDs for its replication rather than inducing synthesis and subsequent degradation. To answer this question, we analyzed the localization of PRRSV particles, the N protein, dsRNA and RTC components with LDs and found that the replication and assembly of PRRSV did not depend on LDs (Fig. 4B through D; Fig. S4A). Combined with the ability of YY1 to promote LD synthesis, we hypothesize that increased LD synthesis after PRRSV infection is a measure of host resistance to the virus. Our results also prove that PRRSV replication is inhibited when large quantities of exogenously induced lipid droplets are synthesized (Fig. 4E through I), indicating that the reprogramming of LD synthesis by YY1 is a coping strategy for host cells to resist virus invasion. PRRSV GP2a has been reported to target GRP78, a central regulator of the unfolded protein response (UPR), to induce endoplasmic reticulum (ER) stress and create favorable conditions for self-replication (62). Additional studies have shown that the ER stress response increases the ability of LDs to store toxic lipids and unfolded proteins that accumulate in the ER membrane to relieve this organelle (63, 64). Combined with our findings, the ER stress response of host cells induced by PRRSV infection also represents a strategy for improving LD synthesis, relieving stress on the host cells and preventing viral replication.

We further found that YY1 affected LD synthesis by negatively regulating FASN expression and positively regulating PPARγ expression (Fig. 5; Fig. S5). FASN is a multienzyme complex composed of seven functionally distinct enzymes and one acyl carrier protein (ACP) that plays a vital role in fatty acid biosynthesis (65, 66). Efficient viral replication in host cells is closely related to FASN-mediated fatty acid synthesis. DENV nonstructural protein 3 (NS3) redistributes FASN to sites of viral replication and increases cellular fatty acid synthesis (67). HCV nonstructural protein 5B (NS5B) interacts with FASN to enhance RNA-dependent RNA polymerase (RdRp) activity and subsequently facilitate HCV replication (68). Whether there is a direct interaction between PRRSV and FASN is unknown, but it has been reported that palmitoylation of the envelope membrane proteins GP5 and M of PRRSV is essential for virus growth (9). Palmitoylation is the posttranslational attachment of fatty acids to cysteine (Cys) residues of membrane proteins (69). Consistent with our findings, FASN knockout reduced the viral titers of PRRSV progeny by 2 logs (Fig. 6D), but this inhibitory effect on PRRSV replication could be reversed by exogenously added palmitic acid (Fig. 6G and H). As a central regulator of lipid metabolism, FASN plays a critical role in the growth of tumors with lipogenic phenotypes, such as those of breast cancer, ovarian cancer, and hepatocellular carcinoma. Several FASN inhibitors, including cerulenin, orlistat, C75, fasnall, and TVB-2640, have been tested against cancer in preclinical studies (70). If a good treatment effect can be achieved, it will provide a reference for preventing and controlling PRRSV.

PPARγ is a member of the peroxisome proliferation-activated receptor family. It promotes fatty acid uptake, triglyceride formation, and lipid storage, thereby increasing insulin sensitivity and glucose metabolism (51, 71). The current view is that LDs support infection, providing microorganisms (such as parasites, bacteria, and viruses) with substrates for effective growth (72). However, our study showed that PPARγ activation promoted intracellular LD synthesis but significantly inhibited PRRSV replication, whereas PPARγ knockout had the opposite effect (Fig. 7). Other factors may also exist, except that the FFAs required for PRRSV replication are taken into LDs for storage. There is limited evidence that proteins localized on mammalian LDs have antibacterial capabilities. Thus, LDs actively participate in innate defence (73). For example, viperin has antiviral activity against HCV and DENV assembled on LDs, histones increase the survival of bacterially challenged Drosophila embryos, and IFN-γ-inducible guanosine triphosphatase (IGTP) is required for resistance to *Toxoplasma gondii* (61, 74, 75). However, because of the important role of PPARγ in LD synthesis and the potential function of LDs in the innate immune system, it is worth developing drugs targeting PPARγ to treat PRRSV infection.

**Fig 8:**
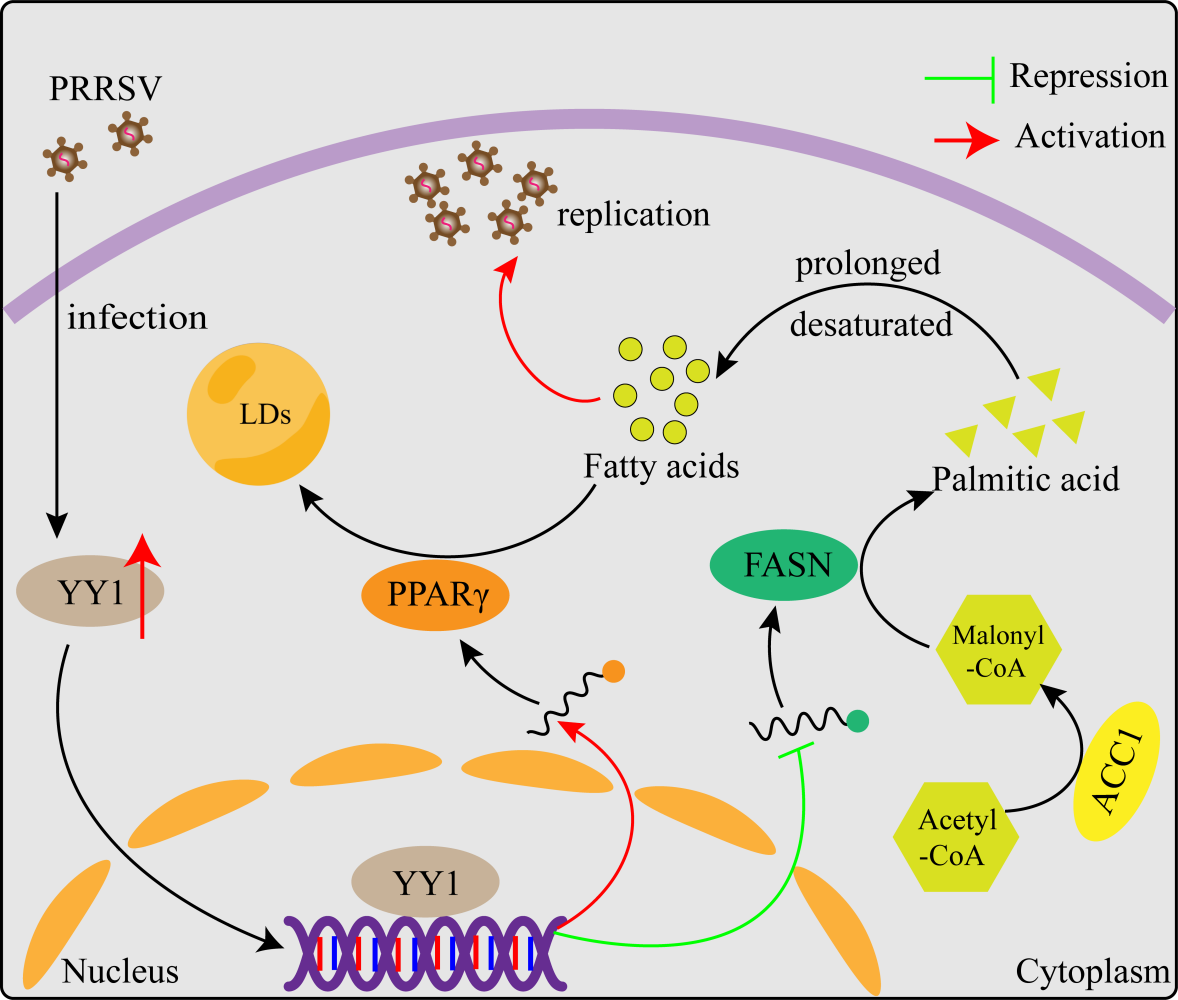
Schematic model for the mechanisms by which YY1 inhibits PRRSV replication. Intracellular FFAs are required for PRRSV replication. On PRRSV infection, the expression of the host transcription factor YY1 is increased. YY1 promotes the synthesis of intracellular LDs and reduces the synthesis of intracellular FFAs by positively regulating the expression of PPARγ and negatively regulating the expression of FASN, thereby inhibiting PRRSV replication.

We found for the first time that PRRSV infection increases the expression level of the host transcription factor YY1 and that YY1-regulated IFN-β is not the main mechanism by which PRRSV replication is inhibited. We further revealed that YY1 promotes the synthesis of intracellular LDs and reduces the synthesis of intracellular FFAs by positively regulating the expression of PPARγ and negatively regulating the expression of FASN, thereby inhibiting PRRSV replication (Fig. 8). In summary, our study provides novel insight showing that host cells reprogram lipid droplet synthesis through YY1 to resist PRRSV infection and suggests that YY1 could be a new therapeutic target for PRRS.

## MATERIALS AND METHODS

### Cells and viruses

PAMs were isolated from 4- to 6-week-old PRRSV-negative pigs as previously described (76) and maintained in RPMI 1640 medium (Gibco, Thermo Fisher Scientific, Inc.) supplemented with 10% fetal bovine serum (FBS) (TransGen Biotech) and penicillin-streptomycin. MARC-145 and HEK-293T cells were cultured in DMEM (Gibco, Thermo Fisher Scientific, Inc.) supplemented with 10% FBS. All cells were grown at 37°C with humidity and 5% CO_2_. A highly pathogenic PRRSV (HP-PRRSV) strain, GD-HD (GenBank ID: KP793736.1), was used in this study. The recombinant PRRSV-mCherry strain was constructed by our laboratory (Lanzhou Veterinary Research Institute, Chinese Academy of Agricultural Sciences, Lanzhou, China). Sendai virus (SeV) was kindly provided by Professor Huaji Qiu (Harbin Veterinary Research Institute, Chinese Academy of Agricultural Sciences).

### Antibodies and chemicals

Anti-YY1 monoclonal antibody (#46395) was purchased from Cell Signaling Technology; anti-FASN monoclonal antibody (#D190620) was purchased from Sangon Biotech; anti-PPARγ polyclonal antibody (#340844) was purchased from ZEN-BIOSCIENCE; anti-IFNAR1 monoclonal antibody (#CY6733); anti-Flag monoclonal antibody (#AB0008) was purchased from Abways Technology; anti-tubulin monoclonal antibody (#HC-101) was purchased from TransGen Biotech; anti-dsRNA monoclonal antibody was purchased from English & Scientific Consulting; anti-PRRSV N monoclonal antibody, anti-PRRSV nsp2 polyclonal antibody, anti-PRRSV nsp9 polyclonal antibody, anti-PRRSV nsp10 polyclonal antibody were prepared by our laboratory; Oil Red O (#O0625), malonyl-CoA (#63410) and Poly(I:C) (#P1530) were purchased from Sigma‒Aldrich; Palmitic acid (#HY-N0830) was purchased from MedChemExpress; Lipofectamine RNAiMAX Transfection Reagent (#13778150) and Lipofectamine 3000 Transfection Reagent (#L3000015) were purchased from Thermo Fisher Scientific; Dual-Luciferase Reporter Assay System Assay Kit (#E1910) was purchased from Promega.

### RNA interference assay

Small interfering RNAs (siRNAs) against YY1 and FASN and negative control (NC) siRNA were designed and synthesized by Tsingke Biotechnology, and the sequences of the siRNAs used are listed in Table 1. MARC-145 cells were seeded in 12-well plates at 1 × 10^5^ cells/well, and PAMs were seeded in 12-well plates at 1 × 10^6^ cells/well and then transfected with the indicated siRNAs at a final concentration of 50 nM using Lipofectamine RNAiMAX according to the manufacturer’s instructions. The effects of YY1, FASN, and PPARγ knockdown were detected by reverse transcriptase PCR (RT-qPCR) and Western blotting.

**TABLE 1:** List of siRNA and sgRNA sequences

Primers	Sequence(5'→3')
si-pYY1-sense	CCGAGUACAUGACAGGAAA
si-pYY1-antisense	UUUCCUGUCAUGUACUCGG
si-mYY1-1#-sense	GCAGAAUUUGCUAGAAUGA
si-mYY1-1#-antisense	UCAUUCUAGCAAAUUCUGC
si-mYY1-2#-sense	CGACACCAACUGGUUCAUA
si-mYY1-2#-antisense	UAUGAACCAGUUGGUGUCG
si-mFASN-sense	GCCGAGUACAAUGUCAACA
si-mFASN-antisense	UGUUGACAUUGUACUCGGC
si-pFASN-sense	GAUCCACCAAGUCGAACAU
si-pFASN-antisense	AUGUUCGACUUGGUGGAUC
si-pPPARγ-sense	UUCCGGAGGACUAUCAGAU
si-pPPARγ-antisense	AUCUGAUAGUCCUCCGGAA
sg-mYY1-sense	CACCGTCGGGTCGTCGGTGACCAG
sg-mYY1-antisense	AAACCTGGTCACCGACGACCCGAC
sg-mIFNAR1-sense	CACCGGGCGCGACGACCCTAGTGC
sg-mIFNAR1-antisense	AAACGGGCGCGACGACCCTAGTGC
sg-mFASN-sense	CACCGTTACGGATGATGACCGTCGC
sg-mFASN-antisense	AAACGCGACGGTCATCATCCGTAAC
sg-mPPARγ-sense	CACCGGGCTGCCAGTTTCGCTCCG
sg-mPPARγ-antisense	AAACCGGAGCGAAACTGGCAGCCC

### Quantitative reverse transcriptase PCR

RT-qPCR was performed as described previously with minor modifications (77). According to the manufacturer’s instructions, total RNA was isolated using TRIzol reagent and reverse transcribed using a PrimeScript RT reagent kit (TaKaRa). RT-qPCR analysis was performed using ChamQ SYBR qPCR Master Mix (Vazyme), and the relative expression levels were calculated by the 2^−ΔΔCT^ method. The primers used are listed in Table 2. β-Actin was used as an internal reference in MARC-145 cells, while HPRT1 was used as an internal reference in PAMs.

**TABLE 2:** List of primers used in the RT-qPCR analysis

Primers	Sequence (5'→3')
pYY1-qF	GAAGATGATGCTCCAAGAAC
pYY1-qR	TCTCCAGTATGAACCAGTTG
mYY1-qF	GAAGATGATGCTCCAAGAAC
mYY1-qR	TCTCCAGTATGAACCAGTTG
mIFNβ-qF	AGGACGCTGCATTGACCAT
mIFNβ-qR	AGCAAGGAGTTTCTCCACAAT
mISG15-qF	AGGCAGCGAACTCATCTT
mISG15-qR	CCAGCATCTTCACCTTCAG
mIFIT2-qF	CACCTGGGGAAACTATGCCT
mIFIT2-qR	GGAAAACTTCTCACAGATGTGCT
PRRSV-ORF7-qF	AGATCATCGCCCAACAAAAC
PRRSV-ORF7-qR	GACACAATTGCCGCTCACTA
mPPARγ-qF	TGAGTTCGCTGTGAAGTT
mPPARγ-qR	CAATCTGTCTGAGGTCTGT
mACC1-qF	TAGTCTGCCACGGATCCAGA
mACC1-qR	GGGAGGGATCTCTGAGGGTT
mFASN-qF	CACATCGTTCGAGCAGCATG
mFASN-qR	AATTTCCAGGAAGCGACCGT
mSCD1-qF	AGGGCCCCAATGTATGTGTG
mSCD1-qR	AAAGATGTAAGGCACCCGGG
mATGL-qF	GAGATGTGCAAGCAGGGCTA
mATGL-qR	ACTCTCCATGGCCTCATCCT
mHSL-qF	CCTCCGGGAGTATGTTACGC
mHSL-qR	ACACCAGCCCAATGGAGATG
mMGLL-qF	GTCTTCCTTCTGGGCCACTC
mMGLL-qR	GTTGAGCACTTTCGCAGCAA
mCPT1A-qF	CTGTATGTCCTTCCAACTCA
mCPT1A-qR	GATGTGCTTGCTGTCTCT
mLPIN1-qF	CAAGCAAGTAGGAGTGTCT
mLPIN1-qR	GCGGAGGCAGAATGAATA
mACSL5-qF	CCGAATGGAACTCTGAAGA
mACSL5-qR	TACTAAGGATGACCGTAAGC
mACADS-qF	TCCAGGTCATCCAGTTCA
mACADS-qR	GGCTTCTTGTTATCCTTCAG
pHPRT1-qF	TGGAAAGAATGTCTTGATTGTTGAAG
pHPRT1-qR	ATCTTTGGATTATGCTGCTTGACC
mβ-Actin -qF	TCCCTGGAGAAGAGCTACGA
mβ-Actin-qR	AGCACTGTGTTGGCGTACAG

### Western blot

Western blotting was performed as described previously with minor modifications (78), and the cells were harvested and lysed in RIPA buffer (Solarbio) containing a protease inhibitor mixture. The cell lysates were separated by 12% SDS‒PAGE and transferred onto PVDF membranes. The membranes were blocked with 5% nonfat milk in PBST for 2 h at room temperature and then incubated with the primary antibody at 4°C overnight. The membranes were washed with PBST and then incubated with a secondary antibody for 1 h at room temperature. After washing, the target proteins were detected with an enhanced chemiluminescence (ECL) kit (Beyotime).

### Immunofluorescence assay

IFA was performed as described previously with the following modifications (79). Cells grown on 24-well plates were fixed with 4% paraformaldehyde (PFA) for 15 min and permeabilized in 0.1% Triton X-100 for 15 min. Then, the cells were incubated in 1% BSA in PBS for 2 h, followed by incubation with primary antibody for 1 h at 37°C. After washing with PBS three times, the cells were incubated with the fluorescent secondary antibody for 1 h. Finally, DAPI was used to stain the nucleus for 5 min. The images were collected by forward fluorescence microscopy and confocal microscopy.

### Viral titration

A 50% tissue culture infective dose (TCID_50_) assay was performed to assess viral titration as described previously with minor modifications (80). MARC-145 cells were seeded into 96-well plates 24 h in advance, and virus supernatants were prepared by 10-fold continuous dilution, and 100 µL volumes of the dilutions were added per well in replicates of eight. The cells were cultured for another 5–6 days, and the TCID_50_ value was calculated by the Reed-Muench method.

### CRISPR-Cas9-mediated knockout in MARC-145 cells

The CRISPR-Cas9 design and analysis method was described in a previous study (81). sgRNAs were ligated into the Lenti-CRISPRv2 plasmid and cotransfected with psPAX2 and pMD2. G into HEK-293T cells to obtain recombinant lentivirus. The recombinant lentiviruses were used to infect MARC-145 cells. Puromycin (8 µg/mL) was added to the cell cultures to select the knockout cells 48 h later. The selected cells were subcloned and inserted into 96-well plates for single-clone growth and analyzed by PCR sequencing and Western blotting. The sequences of the sgRNAs used were as follows: sgYY1-sense, CACCGTCGGGTCGTCGGTGACCAG; sgYY1-antisense, AAACCTGGTCACCGACGACCCGAC; and sgIFNAR1-sense, CACCGGGCGCGA CGACCCTAGTGC, sgIFNAR1-antisense: AAACGGGCGCGACGACCCTAGTGC; sgFASN-sense: CACCGTTACGGATGATGACCGTCGC; sgFASN-antisense: AAACGCGACGGTCATCATCCGTAAC; sgPPARγ-sense: CACCGGGCTGCCAGT TTCGCTCCG, sgPPARγ-antisense: AAACCGGAGCGAAACTGGCAGCCC).

### LD staining

The indicated cells were seeded in 24-well plates at 0.5 × 10^5^ cells/well for 36 h, and the cells were washed with PBS and fixed in 4% PFA for 30 min. After the cells were washed again with PBS, they were stained with Oil Red O (6 mL of saturated Oil Red O solution, 4 mL of distilled water, mixed well, and filtered) for 15 min. The cells were then washed with 75% alcohol for 5 s to remove background staining, rinsed with distilled water, counterstained with modified Harris hematoxylin solution (Solarbio) for 1 min, washed, mounted, and observed under a light microscope.

The indicated cells were seeded in 24-well plates at 0.5 × 10^5^ cells/well for 36 h, and the cells were washed with PBS and fixed in 4% PFA for 30 min. After the cells were washed again with PBS, they were stained with BODIPY 493/503 (BODIPY was dissolved in DMSO to 2 mg/mL of storage solution and finally diluted to 2 µg/mL of working solution using PBS) for 30 min. The cells were then washed with PBS and stained with DAPI for 5 min, and the images were collected by fluorescence microscopy.

### Dual-luciferase reporter assay

A luciferase reporter assay was performed as described previously with the following modifications (82). For FASN and PPARγ promoter luciferase reporter assays, MARC-145 cells were seeded into 24-well plates at a concentration of 70%, and the indicated promoter plasmid and pRL-TK were cotransfected at a ratio of 100 to 1. Forty-eight h later, the cells were collected, and the relative luciferase activity was tested using a dual-luciferase reporter assay system kit (Promega).

### Animal experiment

Ten four-week-old piglets (free of PRRSV, PRV, PCV2, and CSFV) were randomly divided into two groups, with five piglets in each group. The recombinant lentivirus was concentrated at 10^9^ TU/mL according to the instructions of the virus concentration kit. The piglets were inoculated intramuscularly (300 µL) and intranasally (300 µL) with recombinant lentivirus expressing YY1 or control lentivirus and reinfected 2 days later at the same dose. Two d after the completion of lentivirus reinfection, 200 µL of each piglet was inoculated intramuscularly, and 200 µL of each piglet was inoculated intranasally with PRRSV GD-HD (10^5.5^ TCID_50_/mL). Serum samples from piglets in each group were collected on 3 and 14 days after PRRSV infection. All piglets were carefully monitored, rectal temperature was recorded daily until the end of the experiment, and clinical symptoms of PRRS were observed.

### Statistical analysis

The data were obtained from at least three independent experiments and statistically analyzed using GraphPad Prism 5. The results are expressed as the means ± standard deviations (SDs). Statistical significance was determined by Student’s *t*-test, and asterisks indicate statistical significance: NS, not significant; *P < 0.05; **P < 0.01; ***P < 0.001.

## SUPPLEMENTAL MATERIAL

Supplemental figures mbio.01549-24-s0001.docx Figures S1 to S7.

ASM does not own the copyrights to Supplemental Material that may be linked to, or accessed through, an article. The authors have granted ASM a non-exclusive, world-wide license to publish the Supplemental Material files. Please contact the corresponding author directly for reuse.
