# Supplementary material for: Host cells reprogram lipid droplet synthesis through YY1 to resist PRRSV infection
Source: mBio. 2024 Jul 2;15(8):e01549-24. doi: 10.1128/mbio.01549-24 (PMC11323570; doi:10.1128/mbio.01549-24)
Supplement: Supplemental figures — Figures S1 to S7. [file mbio.01549-24-s0001.docx]

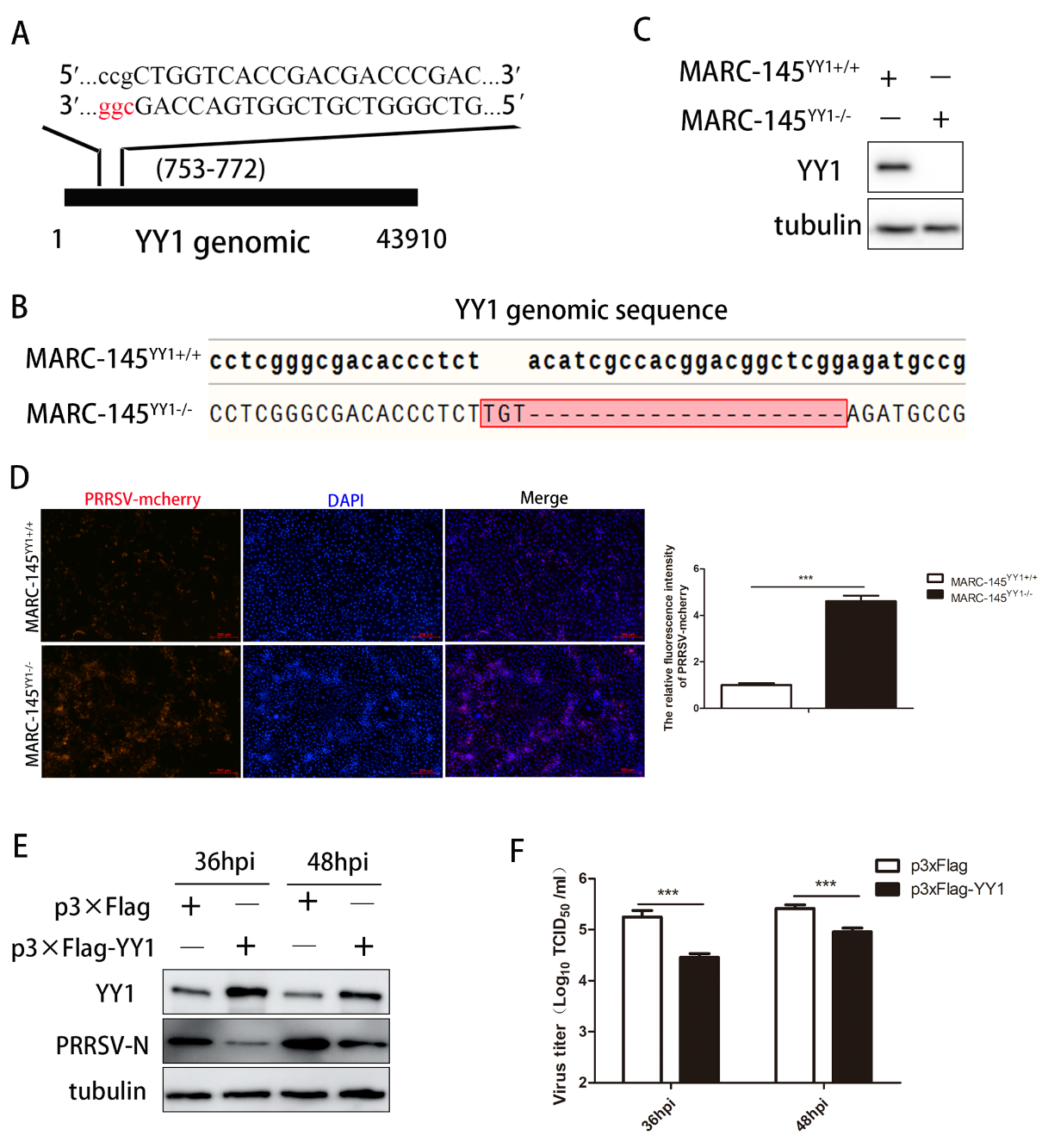


**Supplementary Figure 1.** **YY1 inhibits PRRSV replication *in vitro***

(A) sgRNA design site and sequence of YY1. (B) Identification of YY1 knockout cells by PCR sequencing. (C) Identification of YY1 knockout cells by Western blotting. (D) YY1^+/+^ and YY1^-/-^ MARC-145 cells were infected with PRRSV-mCherry (MOI =1) for 36 h, cells were harvested to determine viral load by immunofluorescence analysis. (E and F) MARC-145 cells were transfected with p3×Flag (vector) or p3×Flag-YY1 for 24 h, followed by infection with PRRSV (MOI =1) for 36 and 48 h, cells and supernatants were harvested to determine (E) YY1 and PRRSV N protein expression, tubulin served as an internal control, and (F) supernatant virus titer. *P* values were calculated using Student's *t*-test. ***, *P*< 0.001.


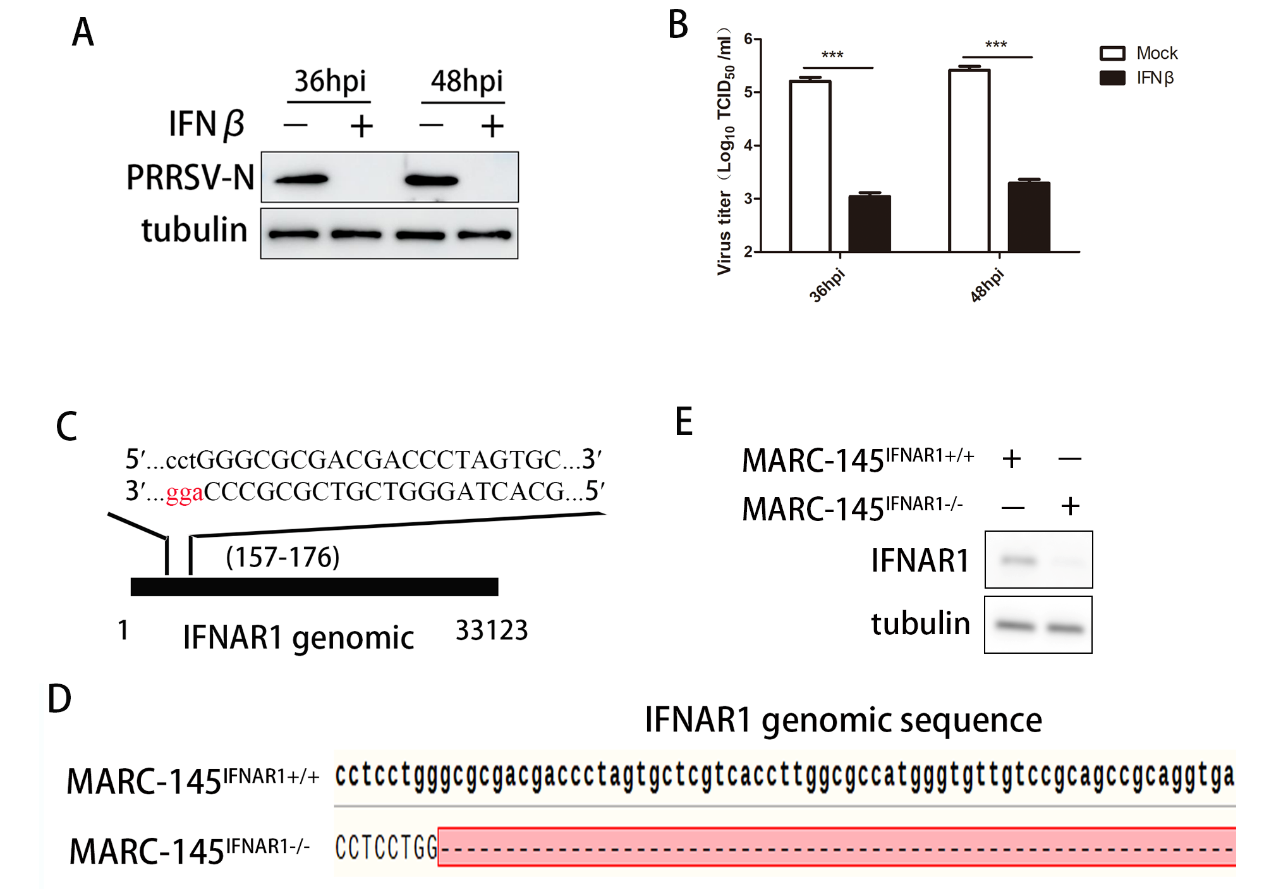


**Supplementary Figure 2. The** **IFNβ inhibits PRRSV replication and construction of IFNAR1 knockout cell line**

(A and B) MARC-145 cells were incubated with recombinant IFNβ at a concentration of 10 ng/ml for 12 h, followed by infection with PRRSV (MOI =1) for the indicated periods. The cells and supernatants were harvested to determine (A) PRRSV N protein expression, tubulin served as an internal control, and (B) supernatant virus titer. (C) sgRNA design site and sequence of IFNAR1. (D) Identification of YY1 knockout cells by PCR sequencing. (E) Identification of IFNAR1 knockout cells by Western blotting. *P* values were calculated using Student's *t*-test. ***, *P*< 0.001.


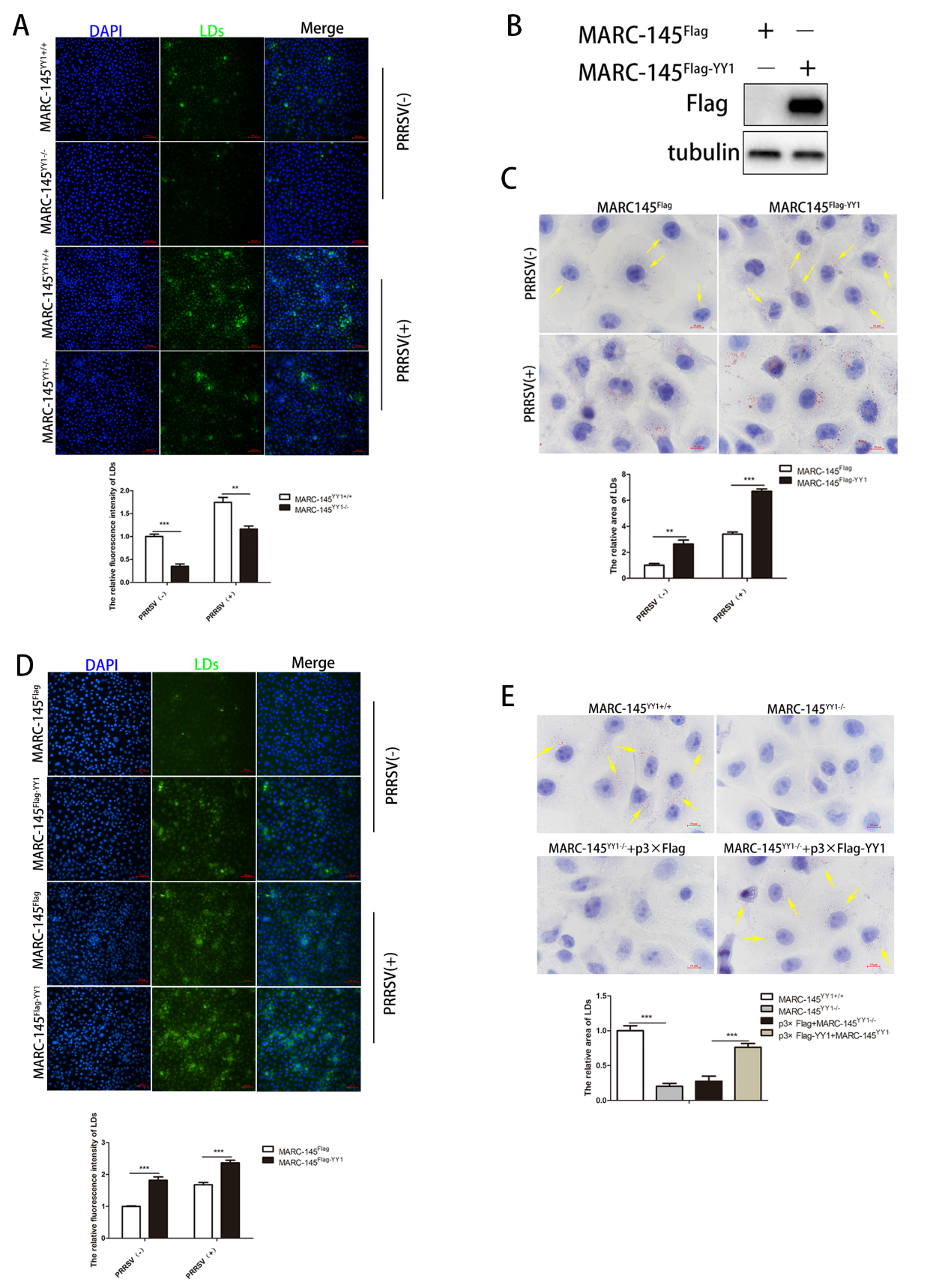


**Supplementary Figure 3.** **YY1 reprograms the synthesis of intracellular lipid droplets**

(A) YY1^+/+^ and YY1^-/-^ MARC-145 cells were fixed and stained the LDs using a BODIPY probe, and nuclei were stained with DAPI. (B) Identification of MARC-145 recombinant cell lines with stably expressing YY1 by Western blotting. (C) MARC-145^Flag^ and MARC-145^Flag-YY1^ cells were fixed and stained the LDs using Oil Red O, and nuclei were counterstained with hematoxylin. (D) MARC-145^Flag^ and MARC-145^Flag-YY1^ cells were fixed and stained the LDs using a BODIPY probe, and nuclei were stained with DAPI. (E) YY1^-/-^ MARC-145 cells were transfected with p3×Flag (vector) or p3×Flag-YY1 for 24 h, cells were fixed and stained the LDs using Oil Red O, and nuclei were counterstained with hematoxylin. *P* values were calculated using Student's *t*-test. **, *P*< 0.01; ***, *P*< 0.001.


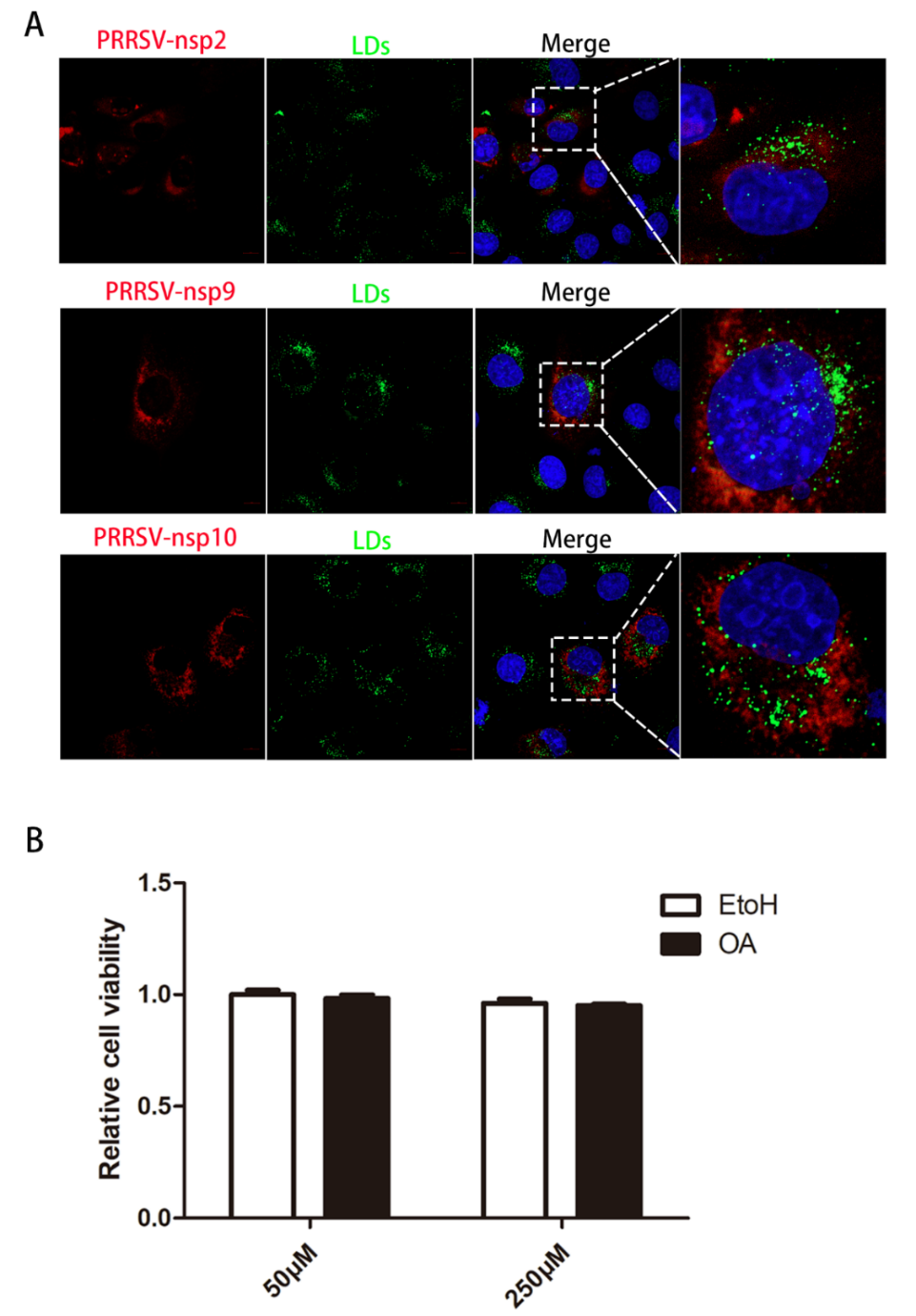


**Supplementary Figure 4.** **PRRSV RTC components nsp2, nsp9 and nsp10 are not co-located with LD and oleic acid on cell activity**

(A) MARC-145 cells were infected with PRRSV (MOI =0.1) for 24 h, cells were stained the LDs using a BODIPY probe (green), labeled the virus using PRRSV nsp2 (red), nsp9 (red) and nsp10 (red) polyclonal antibody respectively, the localization between virus RTC components and LDs. (B) MARC-145 cells were incubated with 50 or 250 μM oleic acid for 24 h, the cell viability was assessed with CCK-8 assay.


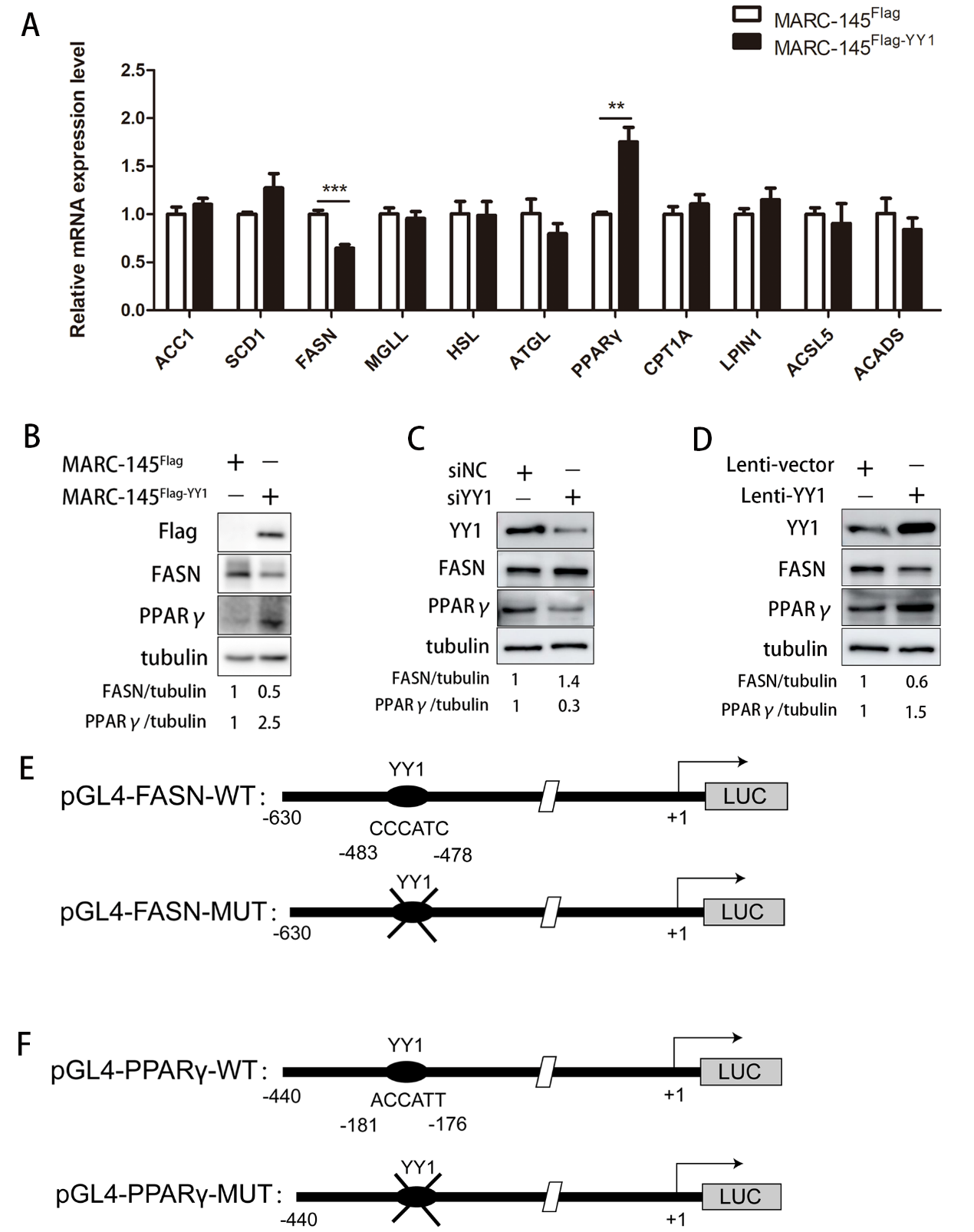


**Supplementary Figure 5.** **YY1 regulates the expression of FASN and PPARγ**

(A) mRNA expression levels of various lipid metabolic-associated factors in MARC-145^Flag^ and MARC-145^Flag-YY1^ cells, as determined using RT-qPCR. (B) Protein expression levels of FASN and PPARγ in MARC-145^Flag^ and MARC-145^Flag-YY1^ cells, as determined using Western blot, tubulin served as an internal control. (C) PAMs were transfected with si-NC or si-YY1 at a concentration of 50 nM for 24 h, cells were harvested to determine YY1, FASN and PPARγ protein expression, tubulin served as an internal control. (D) PAMs were infected with recombinant lentivirus expressing YY1 or control lentivirus for 24 h, cells were harvested to determine YY1, FASN and PPARγ protein expression, tubulin served as an internal control. (E) Schematic representation of the FASN promoter. The binding site of YY1 has been annotated and constructed into the dual-luciferase reporter plasmid pGL4 with the YY1 consensus site (pGL4-FASN-WT), or without the YY1 consensus site (pGL4-FASN-MUT). (F) Schematic representation of the FASN promoter. The binding site of YY1 has been annotated and constructed into the dual-luciferase reporter plasmid pGL4 with the YY1 consensus site (pGL4-PPARγ-WT), or without the YY1 consensus site (pGL4- PPARγ-MUT). *P* values were calculated using Student's *t*-test. **, *P*< 0.01; ***, *P*< 0.001.


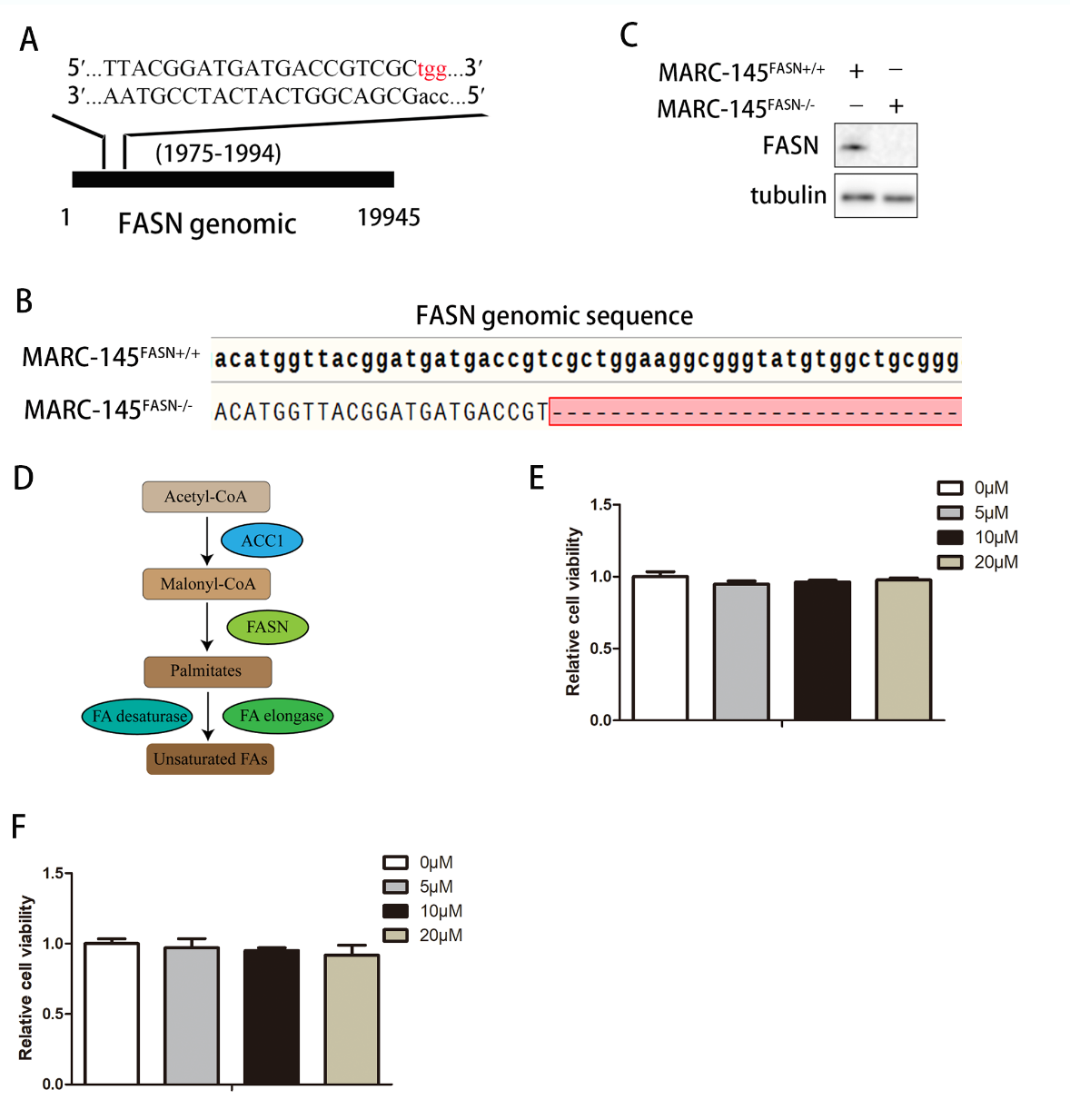


**Supplementary Figure 6.** **Construction of FASN knockout cell line and the effects of malonyl-CoA and palmitic acid on cell activity**

(A) sgRNA design site and sequence of FASN. (B) Identification of FASN knockout cells by PCR sequencing. (C) Identification of FASN knockout cells by Western blot.

(D) Schematic diagram of the fatty acids synthesis. Acetyl-CoA is metabolized to malonyl-CoA by ACC1 and then converted to palmitate by FA synthase (FASN). Palmitic acid is elongated by FA elongases to form saturated fatty acids, which are then metabolized to unsaturated fatty acids by desaturases. (E) MARC-145 cells were incubated with malonyl-CoA (0/5/10/20 μM) for 36 h, the cell viability was assessed with CCK-8 assay. (F) MARC-145 cells were incubated with palmitic acid (0/5/10/20 μM) for 36 h, the cell viability was assessed with CCK-8 assay.


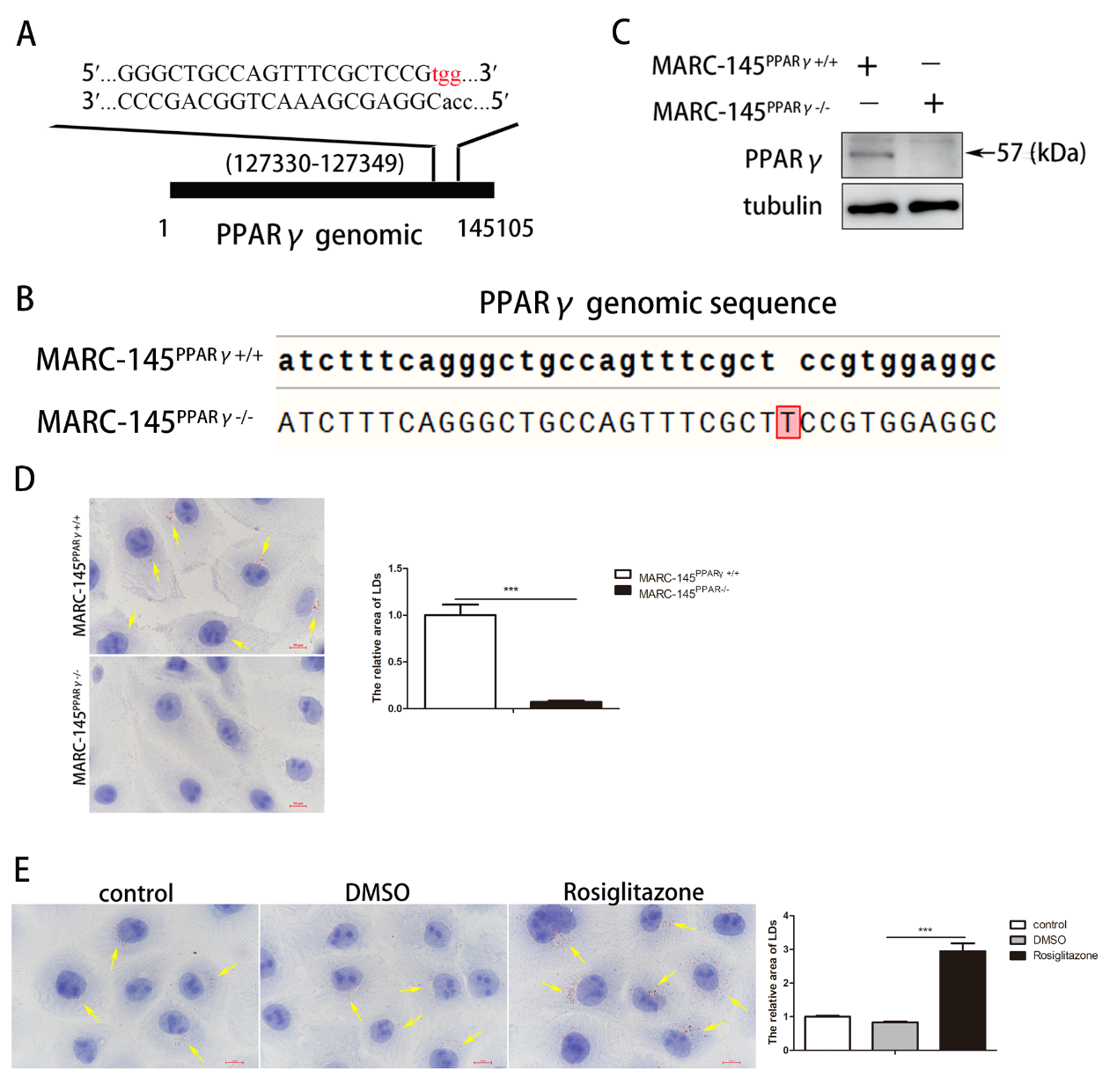
**Supplementary Figure 7.** **Construction of PPARγ knockout cell line and its effect on LD synthesis**

(A) sgRNA design site and sequence of PPARγ. (B) Identification of PPARγ knockout cells by PCR sequencing. (C) Identification of PPARγ knockout cells by Western blot. (D) PPARγ^+/+^ and PPARγ^-/-^ MARC-145 cells were fixed and stained with Oil Red O, and nuclei were counterstained with hematoxylin. (E) MARC-145 cells were incubated with rosiglitazone at a concentration of 10 μM for 36 h, and the cells were fixed and stained with Oil Red O, and nuclei were counterstained with hematoxylin. *P* values were calculated using Student's *t*-test. ***, *P*< 0.001.
